# The synergistic interaction effect between biochar and plant growth-promoting rhizobacteria on beneficial microbial communities in soil

**DOI:** 10.3389/fpls.2024.1501400

**Published:** 2024-12-19

**Authors:** Qianmei Zou, Longyuan Zhao, Lirong Guan, Ping Chen, Jie Zhao, Yueying Zhao, Yunlong Du, Yong Xie

**Affiliations:** ^1^ College of Plant Protection, Yunnan Agricultural University, Kunming, China; ^2^ College of Chemical Engineering, Yunnan Open University, Kunming, China; ^3^ State Key Laboratory for Conservation and Utilization of Bio-Resources in Yunnan, Yunnan Agricultural University, Kunming, China

**Keywords:** beneficial microbial communities, biological agents, rhizosphere microorganisms, soil improvement, soil physicochemical properties

## Abstract

Excessive use of chemical fertilizers and extensive farming can degrade soil properties so that leading to decline in crop yields. Combining plant growth-promoting rhizobacteria (PGPR) with biochar (BC) may be an alternative way to mitigate this situation. However, the proportion of PGPR and BC at which crop yield can be improved, as well as the improvement effect extent on different eco-geographic region and crops, remain unclear. This research used cabbage [*Brassica pekinensis* (Lour.) Rupr.] as the target crop and established as treatment conventional fertilization as a control and a 50% reduction in nitrogen fertilizer at the Yunnan-Guizhou Plateau of China, adding BC or PGPR to evaluate the effects of different treatments on cabbage yield and the soil physicochemical properties. Specifically, high-throughput sequencing probed beneficial soil microbial communities and investigated the impact of BC and PGPR on cabbage yield and soil properties. The results revealed that the soil alkaline hydrolyzable nitrogen (AH-N), available phosphorus (AP), and available potassium (AK) contents were higher in the BC application than in control. The BC application or mixed with PGPR significantly increased the soil organic matter (OM) content (P<0.05), with a maximum of 42.59 g/kg. Further, applying BC or PGPR significantly increased the abundance of beneficial soil microorganisms in the whole growth period of cabbage (P<0.05), such as *Streptomyces*, *Lysobacter*, and *Bacillus*. Meanwhile, the co-application of BC and PGPR increased the abundance of *Pseudomonas*, and also significantly enhanced the Shannon index and Simpson index of bacterial community (P<0.05). Combined or not with PGPR, the BC application significantly enhanced cabbage yield (P<0.05), with the highest yield reached 1.41 fold of the control. Our research indicated that BC is an suitable and promising carrier of PGPR for soil improvement, combining BC and PGPR can effectively ameliorate the diversity of bacterial community even in acid red soil rhizosphere, and the most direct reflection is to improve soil fertility and cabbage yield.

## Graphical Abstract

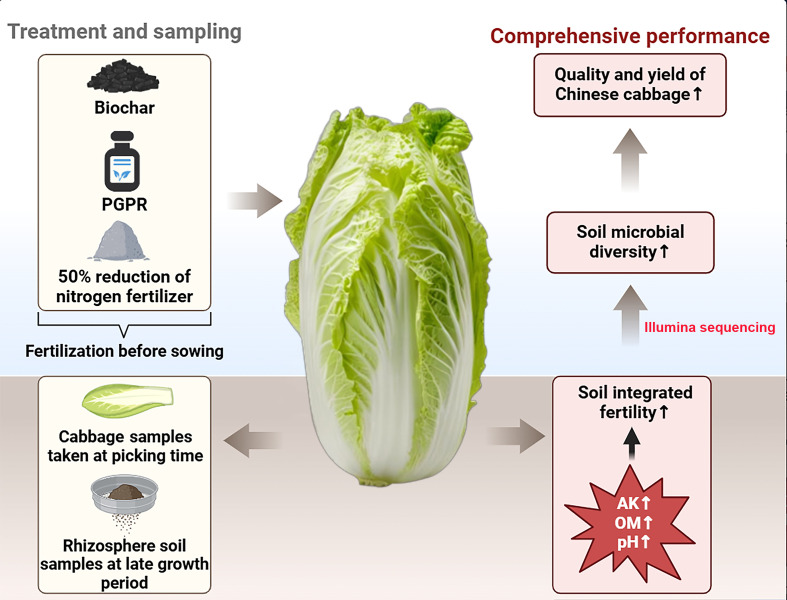

## 1 Introduction

The invention and application of chemical fertilizers are paramount in addressing human food security and ensuring an adequate supply of horticultural products, serving as the material foundation for high-quality and high-yield vegetables. However, due to lack of access to optimizing cultivation techniques and over-pursuing high yield, producers are prone to overusing chemical fertilizers in the vegetable production. Such excessive fertilizer application results in low fertilizer efficiency and poor production benefits, resource waste and product quality declines, and other environmental concern, which adversely impacting the sustainability in agriculture (Lyu et al., 2023). As a result, microbial fertilizers that figuring with improving soil nutrient content, fertilizer utilization and restore soil health have emerged.

Microbial fertilizers contain active microorganisms that can yield specific fertilization effects in agricultural production. Active microorganisms are crucial in generating these effects (Liu et al., 2022). The availability and broad spectrum of plant growth-promoting rhizobacteria (PGPR) are particularly prominent among potential soil microorganisms used for preparing microbial fertilizers. Recently, the application of PGPR in agriculture has steadily increased and is expected to replace chemical fertilizers, pesticides, and other growth regulators, serving as a significant nutritional source for crop growth. Plant growth-promoting rhizobacteria (PGPR) are bacteria that inhabit the rhizosphere of plants and enhance plant growth when inoculated onto seeds, root systems, root tubers, rhizomes, or soil. The mechanism of action of PGPR includes phytohormone production (Park et al., 2017), nitrogen fixation and phosphorus solubilization (Bargaz et al., 2018), inter-organismal signal production (Smith et al., 2015), antibiotic release as biocontrol agents and so on (Ogran et al., 2019). Therefore, research on utilizing PGPR for biological control has become a hot topic (De La Torre-Ruiz et al., 2016). However, studies have also indicated that PGPR may negatively affect plant growth, as cyanides produced by bacteria could adversely impact development (Agbodjato and Babalola, 2024). Moreover, directly incorporating liquid inoculants into the soil may lead to complex exogenous bacteria adhering to soil particles, significantly reducing their vertical mobility and capacity to colonize the rhizosphere (Ahmed et al., 2023). Therefore, adding carriers can enhance the colonization capacity and effectiveness of microorganisms during plant growth and development (Ehinmitan et al., 2024). Thus, exploring colonization carriers or supporting technologies that favor beneficial microbial has become a direction for further promoting microbial fertilizers.

Biochar (BC) is a pyrolytic product derived from organic matter under high-temperature and oxygen-limited conditions. BC possesses an excellent porous structure, and it can improve soil physicochemical properties and enhance soil fertility (Sui et al., 2021). This high-temperature carbon adsorbs non-polar or weakly polar organic solutes, particularly those containing aromatic structures, including enzymes and other substances essential for microbial processes in soil. Research indicates that BC can alter the composition and abundance of soil microorganism communities, suggesting that it is beneficial for the growth of indigenous soil microorganisms (Tan et al., 2022). Besides, BC significantly influences microorganism-mediated soil nutrient transformations, affecting soil structure or nutrient cycling, directly or indirectly impacting plant growth. For instance, BC-based rhizobial inoculants can remarkably enhance the symbiotic relationship between legumes and rhizobia, reducing nitrogen fertilizer requirements and promoting crop growth (Egamberdieva et al., 2016).

Furthermore, studies have shown that the use of BC as a carrier material for microorganism inoculation can provide survival niches and nutrients for microorganism proliferation, safeguarding microorganism from various external stressors, such as desiccation, high temperatures, and toxic elements (Bolan et al., 2023). Biochar exhibits stable properties and has a long residence time in the soil, making its use as a soil amendment a tripartite win strategy influencing crop yield, soil carbon-nitrogen transformation, and global warming potential (Hossain et al., 2020). Thus, we hypothesized that utilizing BC can improve the survival rate of inoculants after entering the soil as a carrier for PGPR and enhance soil properties.

Plant growth-promoting rhizobacteria acted as alive agent, its effects, especially the BC and PGPR synergistic interaction output may varies on different geographic and crop conditions. Currently, research on the co-application of BC and PGPR primarily focuses on drought resistance and productivity effects in food crops such as wheat (*Triticum aestivum* L.) and peanuts (*Arachis hypogaea* L.) (Alam et al., 2024; Noureen et al., 2024), with few of studies focusing on vegetable, especially foliage vegetable (e.g cabbage [*Brassica pekinensis* (Lour.) Rupr.]) which characterizing short-period growth and high multiple cropping index pattern. As a result, excessive use of fertilizers has become a conventional measurement in the production of such horticulture crops. Reasonably, it is sound that reducing fertilizer application is a instant and effective strategy to mitigate adverse consequences. However, this approach may lead to challenges such as nutrient deficiency in the soil and decreased crop yields. Therefore, the purpose of the present research is to elucidate whether the co-application of BC and PGPR can effectively compensate for the adverse effects of fertilizer reduction in the specific environment as the Yunnan-Guizhou Plateau. Specifically, we attempt to shed light on whether the combination can effectively enrich beneficial microorganisms in the soil to improve soil health condition, and whether they have synergistic effects in improving vegetable production. Thus, we employed a nitrogen fertilizer reduction approach to improve soil in conjunction with BC and microbial formulation applications. The effectiveness of BC and PGPR in enhancing soil microorganism communities was determined by comparing soil physicochemical properties, the structure of soil microorganism communities, and crop yields under different treatments. The present study aims to provide a theoretical basis for the effective application of PGPR in agricultural/horticultural production.

## 2 Materials and methods

### 2.1 Overview of the experimental area

The experimental area was set at Taixing Agricultural Technology Co., Ltd. Vegetable Production Base, Dali City, Xiangyun County, Yunnan Province (25°12’05” N,100°25’22” E). The experimental area is located in northwest central Yunnan Province. The area has a northern subtropical monsoon climate of the plateau, with an average annual temperature of 14.7°C, a frost-free period of 228 days, an annual rainfall of 810 mm, and an annual sunshine duration of 2,624 h. Initially, an analysis of soil physicochemical properties was conducted, the relevant index are as follows: the contents of soil pH (pH), organic matter (OM), alkaline hydrolyzable nitrogen (AH-N), available phosphorus (AP), and available potassium (AK) are 7.60, 26.59 mg/kg, 90.30 mg/kg, 27.97 mg/kg, respectively.

### 2.2 Materials

The tested crop was cabbage [*Brassica pekinensis* (Lour.) Rupr.], and the seeds were supplied by the Taixing Agricultural Technology Co., Ltd. The fertilizers applied, sources and their nutrient content are summarized in **Supplementary Table 1**. The BC treatment was formulated using a 3:2 mass ratio of BC to lignite, and the biological agents were granular PGPR (**Supplementary Table 2**).

### 2.3 Experimental design

Six treatments and one control were designed in this experiment (**Table 1**). A completely randomized experimental design was adopted, repeated three times, with a plot area of 14 m^2^, totaling 21 residential areas. The planted density of Chinese cabbage was 25 cm × 25 cm, totaling eight rows (28 plants per row) and 28 lines (eight plants per line).

**Table 1:** Field trails design and treatments in this research.

Treatment	Experimental design			
	BC(kg/hm^2)^	Lignite (kg/hm^2^)	PGPR (kg/hm^2^)	Reduction in nitrogen fertilizer (%)
A	0	0	0	0
B	9.00 × 10^3^	6.00 × 10^3^	0	0
C	0	0	1.50 × 10^2^	0
D	9.00 × 10^3^	6.00 × 10^3^	1.50 × 10^2^	0
E	9.00 × 10^3^	6.00 × 10^3^	0	50
F	0	0	1.50 × 10^2^	50
G	9.00 × 10^3^	6.00 × 10^3^	1.50 × 10^2^	50

Two-season cabbage was planted to avoid the influence of temperature and light conditions across different seasons. Biochar, lignite, and PGPR were sprayed and plowed evenly before planting cabbage. The fertilization schemes for each treatment are detailed in **Supplementary Table 3**. The control and conventional fertilization amounts of nitrogen, phosphorus, and potassium were 347.70 kg/hm^2^, 86.25 kg/hm^2^, 123.00 kg/hm^2^. The nitrogen, phosphorus, and potassium amounts in the nitrogen reduction treatment were 174.75 kg/hm^2^, 79.20 kg/hm^2^, 173.10 kg/hm^2^. All treatments used a water fertilization mixture, and the reduction ratios were respectively 50%, 8%, and -41%.

### 2.4 Methods

#### 2.4.1 Sample collection

Before the two-season cabbage planting, soil samples before planting cabbage (CK) and soil samples adding microbial agents to the soil before planting cabbage (CK-0) were collected using the five-point sampling method as blank controls for measuring the soil microbial community structure. Soil samples of 0–20 cm depth were collected each time. These samples were collected from each plot repeatedly three times and were mixed. A total of six rhizosphere samples were collected each season. Five cabbage plants with the same growth vigor were determined using an “S”-shaped point arrangement in the middle and late growth periods of the two seasons of cabbage. Next, the weeds and dead leaves around the cabbages were cleaned. Later, the cabbage plants were uprooted, and after shaking off the larger pieces of soil from the root system, small pieces of soil or soil particles attached to the root surface (about 2–3 mm) were collected as rhizosphere soil samples (Lu et al., 2015), soil samples in the same plot were evenly mixed as a mixed sample, and 21 rhizosphere samples were collected in each period. A total of 42 rhizosphere samples were collected in each season. Some mixed soil samples were air dried and stored to determine soil physicochemical properties. In contrast, others were stored in a refrigerator at −80°C to extract the total deoxyribonucleic acid (DNA) of microorganisms for high-throughput sequencing.

Five additional plants collected in each plot were weighed during the late growth of the two-season cabbage. The number of missing and variant plants in the plot was investigated. The actual final harvested plants in the plot were determined, and the entire harvest was obtained as the yield.

#### 2.4.2 Cabbage yield

The actual yield during the harvesting of Chinese cabbage was measured for each plot and converted to yield per unit area using the equation below:


$$Y_{p}\left(kg/hm^{2}\right)=\frac{A_{y}\left(kg\right)}{A_{p}\left(m^{2}\right)}\times 10^{3}$$

(*Y_p_* represents the yield per unit area; *A_y_* represents the actual yield per plot; *A_p_* represents the area per plot).

#### 2.4.3 Soil physicochemical properties

The available nutrient content in the soil was determined after drying the sampled soil using the following methods: Soil pH was determined at a 1:5 (w/v) soil/distilled water ratio using a pH meter (Mettler Toledo Delta 320). Soil OM was determined using the potassium dichromate external heating method. Soil AH-N was measured using the alkaline hydrolysis diffusion method. Soil AP was measured using molybdenum antimony colorimetry. Soil AK was measured using the flame photometry method (Klotz et al., 2023).

#### 2.4.4 High-throughput sequencing of soil bacteria

The Illumina sequencing technology was used for high-throughput sequencing. Soil DNA was extracted using a DNA extraction solution. The DNA concentration was detected using agarose gel electrophoresis and NanoDrop2000 (Thermo Scientific, Wilmington, USA). The V4 region of bacterial 16S ribosomal ribonucleic acid (rRNA) genes was amplified using the primers 515F (5’-GTGYCAGCMGCCGCGGTAA-3’) and 806R(5’-GGACTACHVGGGTWTCTAAT -3’) with genomic DNA as a template. The PCR products were quantified using Qubit 2.0 Fluorometer (Thermo Scientific, Wilmington, USA) based on the preliminary results from electrophoresis. Then, according to the sequencing requirements of each sample, the corresponding proportions were mixed. Using the PE250 sequencing method, the raw offline data obtained from sequencing were concatenated and filtered to obtain high-quality target sequences for subsequent analysis.

### 2.5 Statistical analysis of data

Field experiment and laboratory test data were preliminarily organized and drawn using Excel 2013. Using SPSS 19.0, conducted variance analysis and significance tests on soil physicochemical properties and cabbage yield. GraphPad Prism 8.0.2 and R software were used for beta diversity analyses and graphic analysis.

Based on the Usearch software (http://drive5.com/uparse/), the UPARSE algorithm was utilized for OTU clustering at a consistency level of 97%. The sequence with the highest frequency within each OTU was selected as the representative sequence. Taxonomic annotation analysis was conducted using the UCLUST classification method in conjunction with the SILVA database (Release_123 http://www.arb-silva.de/). Representative sequences underwent multiple sequence alignment via PyNAST. Additionally, a phylogenetic tree was constructed using FastTree. Use R language for various data conversions and use ggplot2 package for bacterial community composition analysis.

Alpha diversity analysis was performed using R language. Wilcoxon rank sum test and Kruskal-Wallis rank sum test were performed, and multiple comparisons were also performed. Beta diversity analysis was performed using R language. The Unifrac distance, Bray-Curtis and Jaccard distances were calculated. PCA analysis was performed using vegan package.

## 3 Results

### 3.1 Effect of different fertilization treatments on soil physicochemical properties

Adding BC and PGPR increased the AH-N content. The BC application in the E treatment significantly increased the AP content at 160 days, reaching 19.49 mg/kg. In contrast, the improvement effect was not significant among the other treatments. Treatments D, B, and F increased pH that reached 7.78, indicating that BC and PGPR have an upward-regulating effect on pH. The F treatment reached the highest AK content at 60 days (389.95 mg/kg). The F and G treatments reached the highest content after 160 days. This result indicated that the PGPR application or a combination of BC and PGPR enhanced the AK content. Treatment D reached the highest content at 115 days, and treatment G had the highest content at 160 days, reaching 42.59 g/kg. The results showed that BC and PGPR applications could increase OM content. Still, the effect of applying PGPR alone was smaller than that of applying BC alone or BC in combination with PGPR (**Table 2**).

**Table 2:** Changes in the physicochemical properties of soil under different treatments at the same time.

Physicochemical property	Time/Day	A	B	C	D	E	F	G
AH-N (mg/kg)	30d	138.60 ± 3.21a	139.30 ± 6.10a	105.00 ± 17.94bc	117.60 ± 2.10ab	102.90 ± 3.64bc	107.80 ± 9.72bc	86.10 ± 5.56c
	60d	120.40 ± 19.03a	102.90 ± 29.40a	101.50 ± 6.89a	90.30 ± 12.77a	100.80 ± 16.31a	97.30 ± 3.05a	94.50 ± 9.15a
	115d	128.80 ± 2.52a	128.10 ± 13.66a	149.10 ± 29.87a	133.00 ± 7.41a	128.11 ± 25.66a	155.40 ± 18.35a	112.70 ± 7.41a
	160d	129.50 ± 20.91a	102.90 ± 10.50ab	116.20 ± 1.85ab	121.10 ± 6.89ab	125.30 ± 7.89a	88.90 ± 9.10b	132.30 ± 8.49a
AP (mg/kg)	30d	44.04 ± 9.66a	34.60 ± 8.31a	38.49 ± 15.10a	23.12 ± 5.29a	27.78 ± 6.02a	44.74 ± 0.40a	20.40 ± 6.11a
	60d	9.57 ± 1.80a	8.88 ± 1.48a	8.01 ± 0.81a	9.49 ± 1.59a	10.10 ± 2.23a	8.22 ± 0.50a	8.28 ± 1.78a
	115d	8.94 ± 0.47a	9.46 ± 1.02a	6.93 ± 1.47b	10.10 ± 2.44a	11.77 ± 1.17a	9.46 ± 0.70a	9.59 ± 0.35a
	160d	18.18 ± 3.17ab	18.11 ± 4.67ab	12.26 ± 0.38b	13.08 ± 0.77ab	19.49 ± 2.91a	14.10 ± 4.47ab	14.07 ± 0.41ab
AK (mg/kg)	30d	345.29 ± 22.52a	346.34 ± 15.10a	308.73 ± 19.98a	357.44 ± 2.74a	346.86 ± 20.68a	342.16 ± 22.46a	374.02 ± 22.49a
	60d	305.47 ± 28.99ab	286.93 ± 25.79b	351.56 ± 40.74ab	309.90 ± 26.57ab	345.69 ± 24.74ab	389.95 ± 8.51a	382.64 ± 15.16a
	115d	319.18 ± 21.78a	302.60 ± 9.42a	367.49 ± 36.87a	308.86 ± 18.19a	320.22 ± 15.49a	339.03 ± 14.69a	334.85 ± 11.99a
	160d	264.73 ± 35.42b	289.15 ± 39.04ab	307.43 ± 4.31ab	334.33 ± 34.32ab	344.25 ± 29.81ab	373.24 ± 3.41a	364.10 ± 6.77a
OM (g/kg)	30d	27.89 ± 4.29bc	34.75 ± 3.86abc	25.80 ± 0.96bc	41.69 ± 5.89ab	43.42 ± 3.39a	26.81 ± 6.84bc	34.79 ± 5.17abc
	60d	29.08 ± 3.60a	39.90 ± 0.51a	23.66 ± 4.03a	41.36 ± 4.41a	35.72 ± 7.03a	24.71 ± 5.31a	32.86 ± 8.89a
	115d	35.86 ± 2.90c	71.74 ± 4.63b	45.25 ± 12.90c	102.05 ± 2.42a	80.53 ± 2.45b	34.61 ± 4.34c	72.80 ± 6.69b
	160d	34.34 ± 6.10a	31.98 ± 6.79a	28.32 ± 4.96a	39.25 ± 7.44a	41.02 ± 9.85a	24.47 ± 8.20a	42.59 ± 10.12a
pH	30d	7.42 ± 0.09ab	7.22 ± 0.02b	7.36 ± 0.06b	7.62 ± 0.18a	7.58 ± 0.09a	7.44 ± 0.09ab	7.58 ± 0.01a
	60d	7.63 ± 0.14a	7.66 ± 0.01a	7.49 ± 0.07a	7.78 ± 0.14a	7.66 ± 0.02a	7.60 ± 0.03a	7.76 ± 0.03a
	115d	7.57 ± 0.01a	7.58 ± 0.03a	7.55 ± 0.05a	7.51 ± 0.04a	7.47 ± 0.04a	7.45 ± 0.05a	7.56 ± 0.09a
	160d	7.40 ± 0.03b	7.41 ± 0.05b	7.46 ± 0.02ab	7.41 ± 0.03b	7.38 ± 0.06b	7.61 ± 0.06a	7.45 ± 0.06b

The data in the table is the mean ± standard error of 3 replicates. Different lowercase letters in the same row indicate significant differences between treatments (p<0.05), the same as below.

In conclusion, adding BC and PGPR positively impacted soil physicochemical properties, improving soil health and fertility.

### 3.2 Effect of different fertilization treatments on soil microbial community diversity

#### 3.2.1 Analysis of the sequencing structure of rhizosphere soil

A total of 3,183,060 sequences were obtained during the experiment. The final number of tag sequences used for subsequent analysis was 2,849,972 after filtering for chimeras. These tag sequences had an average sequence length of 288 bp. The dilution curves for each soil sample tended to slow gradually, indicating that the sequencing depth contained most bacterial types in the samples (**Supplementary Figure 1**). This result suggests that the sequencing adequacy was appropriate and could be utilized to analyze the community structure of bacteria.

#### 3.2.2 Structure of soil bacterial phylum-level communities

**Figure 1:**
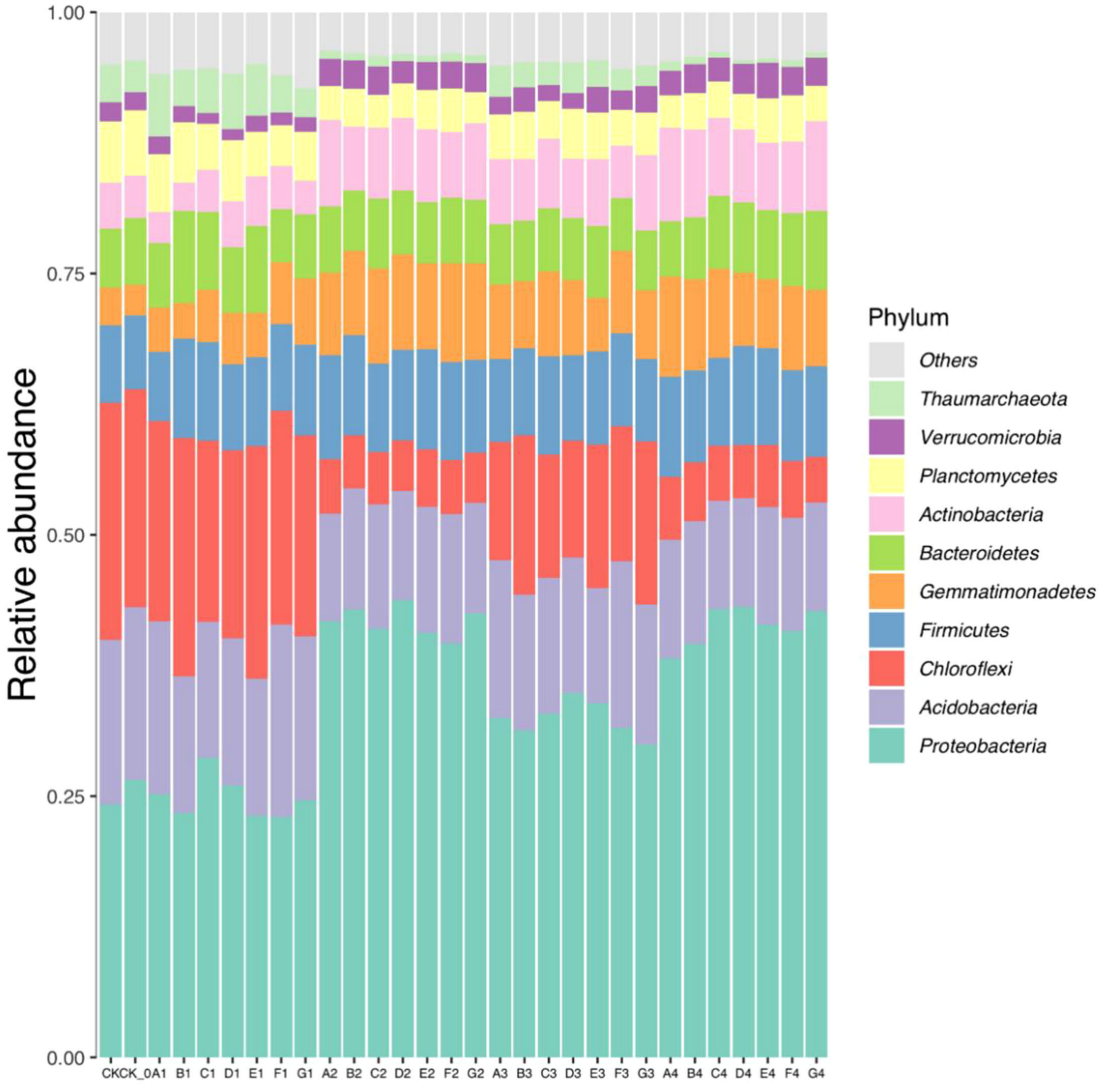
The relative abundance of dominant groups of bacteria at the phylum level in soil samples.

The bacterial operational taxonomic units (OTUs) obtained from sequencing soil samples were classified into 45 phyla, 117 classes, 310 orders, 547 families, and 1167 genera. *Proteobacteria*, *Acidobacteria*, *Chloroflexi*, *Firmicutes*, *Gemmatimonadete*, *Bacteroidetes*, *Actinobacteria*, *Planctomycetes*, *Verrucomicrobia*, and *Thaumarchaeota* were the dominant soil bacteria, and their average relative abundance was greater than 0.70% (**Figure 1**). The relative abundance of *Proteobacteria*, *Gemmatimonadetes*, *Actinobacteria*, *Bacteroidetes*, and *Firmicutes* significantly increased after the cultivation of cabbage compared to the control, whereas *Chloroflexi* and *Thaumarchaeota* decreased.

#### 3.2.3 Structure of soil bacterial genus-level communities

**Figure 2:**
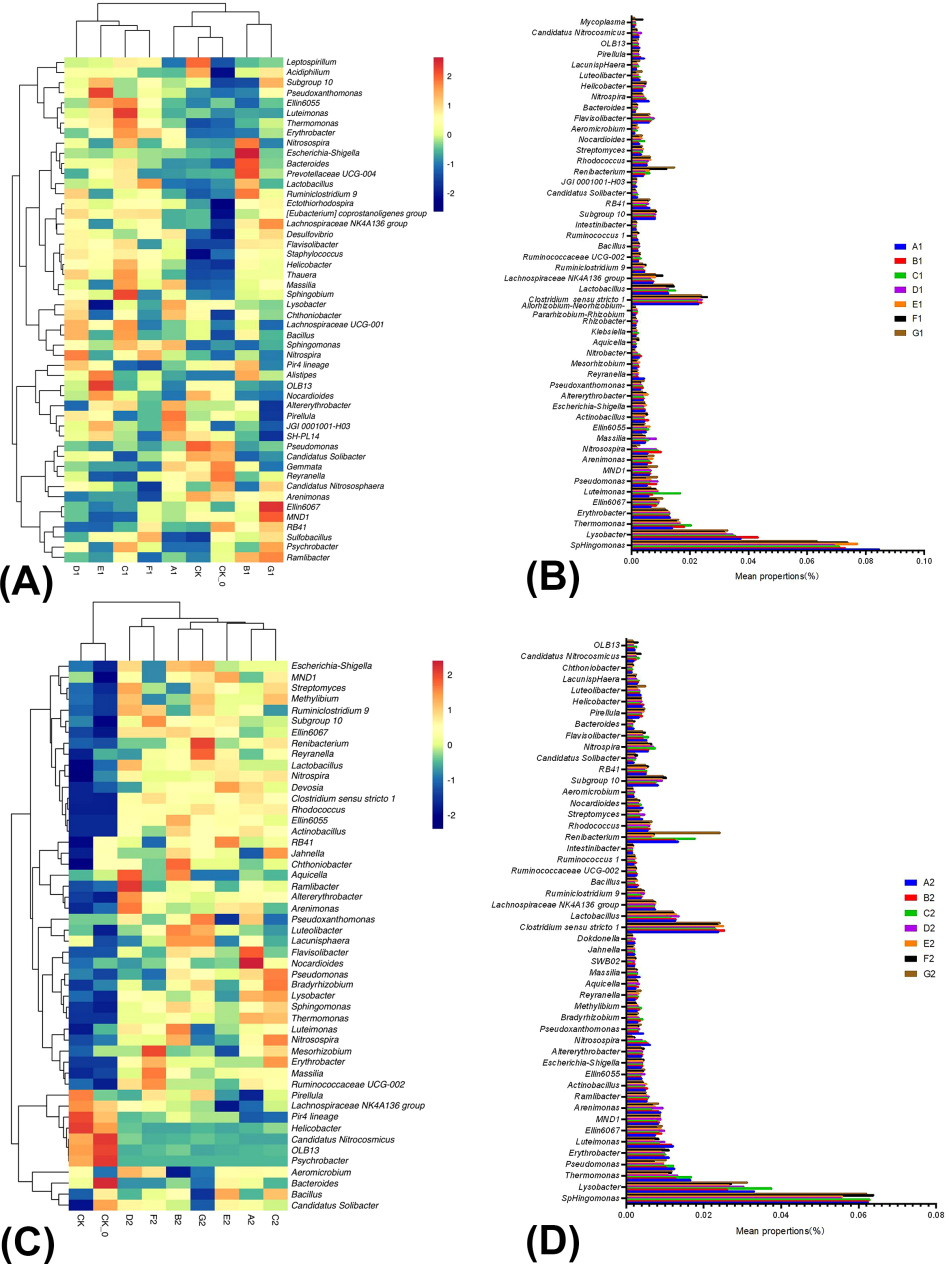
The relative abundance and similarity of bacterial genus level in soil under different treatments in summer season Chinese cabbage and the classification. The panel **(A)** is the relative abundance and similarity of bacterial genus level in soil under different treatments in the mid-growth of summer season Chinese cabbage; The panel **(B)** is the classification of bacteria in the mid-growth of summer season Chinese cabbage; The panel **(C)** is the relative abundance and similarity of bacterial genus-level in soil under different treatments at the late growth of summer season Chinese cabbage; The panel **(D)** is the classification of bacteria in the late growth of summer season Chinese cabbage.

**Figure 2A** illustrated the relative abundance and correlations of bacteria among the nine samples collected during the mid-growth period of cabbage in the summer season. There were significant differences between B1 and G1 and among CK, CK-0, and A1. Significant differences in community structure among C1, F1, E1, and D1 and CK, CK-0, and A1 were also detected. Therefore, the BC or PGPR application altered the soil bacterial community structure (**Figure 2A**). We compared the 50 high-ranking species with high abundance rankings between treatments based on the community abundance data from the samples. At the genus level, the BC and PGPR applications significantly increased the relative abundance of beneficial microorganisms in the soil, including *Nitrosospira* and *Lysobacter*, which have biocontrol functions. *Thermomonas* and *Luteimonas* are associated with antibacterial effects, and *Bacteroides* is related to nutrient cycling. Furthermore, the BC and PGPR applications also significantly reduced the relative abundance of organic pollutant-degrading bacteria *Reyranella*, *Nitrosospira*, and *Nitrobacter*, which are associated with the nitrogen cycling process. Besides, the reduction of nitrogen fertilizer application did not decrease the relative abundance of beneficial bacteria, which is feasible (**Figure 2B**; **Supplementary Table 4**).

**Figure 2C** illustrated the relative abundance and correlations of bacteria among nine samples collected during the late growth period of cabbage in the summer. There were significant differences between the treatments (CK and CK-0 and others) and significant differences in community structure among B2 and G2, D2 and F2, and A2. Applying BC, PGPR, or both combined with reduced nitrogen fertilizer altered the soil community structure. At the genus level of bacteria, applying BC and PGPR significantly increased the relative abundance of *Bacillus*, *Lysobacter*, and *Bradyrhizobium*, all were biocontrol agents that enhanced plant growth and inhibited the growth of pathogenic microorganisms. The abundance of these three microorganisms constituted 0.77% of the total microbial population in the samples, indicating that the application of BC and PGPR can promote plant growth and development and inhibit the occurrence of diseases (**Figure 2D**; **Supplementary Table 5**).

**Figure 3:**
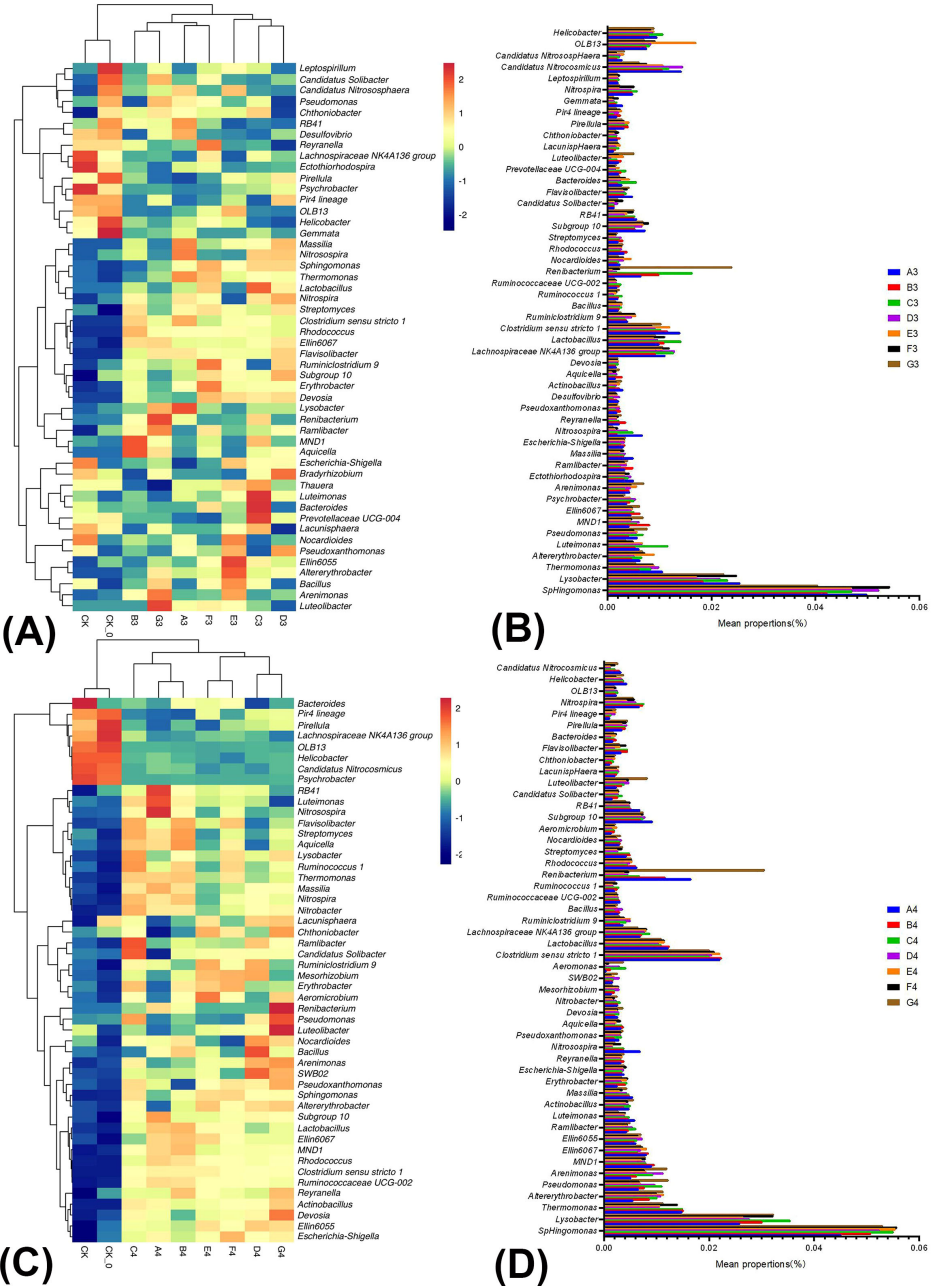
The relative abundance and similarity of bacterial genus level in soil under different treatments in winter season Chinese cabbage and the classification. The panel **(A)** is the relative abundance and similarity of bacterial genus level in soil under different treatments at the mid-growth of winter season Chinese cabbage; The panel **(B)** is the classification of bacteria in the mid-growth of winter season Chinese cabbage; The panel **(C)** is the relative abundance and similarity of bacterial genus-level in soil under different treatments at the late growth of winter season Chinese cabbage; The panel **(D)** is the classification of bacteria in the late growth of winter season Chinese cabbage.

**Figure 3A** illustrated the relative abundance and correlations of bacteria among the nine samples collected during the mid-growth period of cabbage in the winter season. There were significant differences between the treatments (CK, CK-0) and others, and significant differences in community structure between B3, D3, E3, or G3 and A2. This indicated that A3–G3 treatments resulted in substantial changes in the community structure compared to the control. At the genus level, the BC and PGPR application significantly increased the relative abundance of beneficial microorganisms in the soil. For instance, the abundance of *Lactobacillus, Bacillus*, and *Pseudomonas*, which have biocontrol functions, increased, along with that of *Pseudoxanthomonas* and *Streptomyces*, which have antibiotic properties and facilitate rhizosphere colonization. The abundance of *Chthoniobacter* and *Luteolibacter*, which have plant growth-promoting functions, and *Bacteroides* and *Bradyrhizobium*, which participate in the nitrogen cycle and have the nitrogen-fixing capabilities of the genera, also increased (**Figure 3B**; **Supplementary Table 6**). In summary, applying BC and PGPR effectively suppressed diseases, promoted plant growth and development, and enhanced soil quality.

**Figure 3C** illustrated the relative abundance and correlations of bacteria among the nine samples collected during the late growth period of cabbage in winter. There were significant differences between treatments (CK and CK-0). The A4–G4 treatments resulted in substantial alterations in the community structure compared to the control treatment. At the genus level, the BC and PGPR application or their combination with reduced nitrogen fertilization, significantly increased the relative abundance of beneficial microorganisms in the soil. These microorganisms included *Bacillus*, *Pseudomonas*, and *Lactobacillus*, which have biocontrol functions, *Pseudoxanthomonas* and *Streptomyces*, which have antibiotic properties and facilitate rhizosphere colonization, *Chthoniobacter* and *Luteolibacter*, which have a plant growth-promoting function, *Nitrobacter*, *Bacteroides*, and *Bradyrhizobium*, which participate in the nitrogen cycle and have the nitrogen-fixing capabilities of the genera (**Figure 3D**; **Supplementary Table 7**). The BC and PGPR applications or their combination with reduced nitrogen fertilization enhanced soil health and fertility.

#### 3.2.4 Alpha diversity analyses

The Simpson index for the E treatment was lower than the control at 30 days. In contrast, the indexes in other treatments increased compared to control. The indexes across various treatments were not significant difference at 60 days. However, the indexes were the highest for the E treatment at 115 and 160 days (**Table 3**). In summary, the application of BC and PGPR significantly enhanced the richness and diversity of soil microorganisms, with nitrogen reduction combined with BC having a better effect. According to **Supplementary Table 8**, the species richness and community diversity of the treatments increased with the advancement of the growth period of the cabbage. Regarding species dominance, there were no significant differences across various periods under G treatment, indicating that species dominance remained unchanged. Still, other treatments increased with the growth stages of the cabbage (**Supplementary Table 8**).

**Table 3:** Bacterial diversity index comparison of different treatments at the same time.

Time	Treatments	Observed	Chao1	Shannon	Simpson
30d	A	943.33 d	1971.46 b	6.22 c	0.9954 de
	B	1034.33 a	2363.63 a	6.41 a	0.9965 ab
	C	986.33 bc	2162.81 ab	6.30 b	0.9960 bc
	D	967.33 cd	2143.18 ab	6.27 bc	0.9958 cd
	E	975.00 bcd	2021.41 ab	6.25 bc	0.9949 e
	F	941.00 d	2006.25 ab	6.24 c	0.9956 cd
	G	1006.67 ab	2274.97 ab	6.39 a	0.9966 a
60d	A	1035.00 a	2456.52 a	6.42 a	0.9967 a
	B	1029.67 a	2370.26 a	6.40 a	0.9966 a
	C	1034.00 a	2447.25 a	6.40 a	0.9966 a
	D	1020.33 a	2155.77 a	6.41 a	0.9968 a
	E	1066.33 a	2408.25 a	6.49 a	0.9970 a
	F	1018.00 a	2100.30 a	6.44 a	0.9969 a
	G	1014.00 a	2278.52 a	6.41 a	0.9968 a
115d	A	1102.33 a	2612.89 a	6.52 bc	0.9971 bc
	B	1131.33 a	2511.65 a	6.60 ab	0.9975 a
	C	1103.00 a	2730.49 a	6.52 bc	0.9971 bc
	D	1094.00 a	2387.58 a	6.54 bc	0.9973 ab
	E	1167.00 a	2745.82 a	6.65 a	0.9976 a
	F	946.00 b	1731.75 b	6.39 d	0.9969 c
	G	1094.33 a	2404.12 a	6.53 bc	0.9969 c
160d	A	1070.33 c	2417.82 a	6.50 c	0.9970 c
	B	1123.00 ab	2662.78 a	6.57 ab	0.9973 b
	C	1106.33 abc	2583.75 a	6.56 b	0.9974 ab
	D	1114.00 abc	2522.94 a	6.57 ab	0.9975 a
	E	1127.00 ab	2672.02 a	6.60 ab	0.9975 a
	F	1146.67 a	2727.46 a	6.62 a	0.9975 a
	G	1093.67 bc	2556.51 a	6.50 c	0.9967 d

Different lowercase letters in the same row indicate significant differences between treatments (p<0.05).

#### 3.2.5 Beta diversity analyses

**Figure 4:**
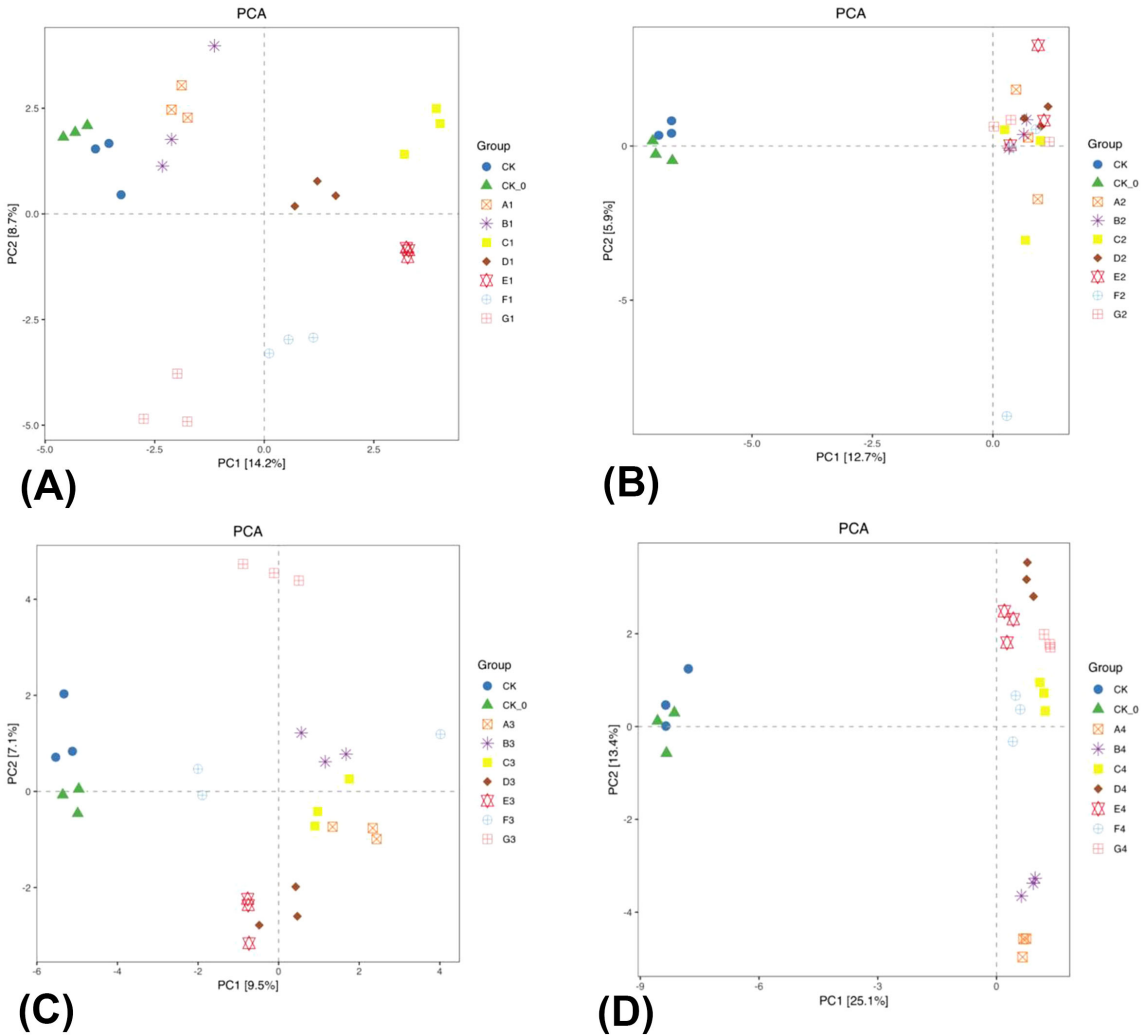
Principal component analysis (PCA) two-dimensional diagram of bacterial β-diversity in rhizosphere soil of the two season Chinese cabbage. The panel **(A)** is the PCA analysis map of the mid-growth period of the summer season of Chinese cabbage; The panel **(B)** is the PCA analysis map of the late growth period of the summer season Chinese cabbage; The panel **(C)** is the PCA analysis map of the mid-growth period of the winter season of Chinese cabbage; The panel **(D)** is the PCA analysis map of the late growth period of the winter season of Chinese cabbage.

A PCA was conducted to compare the CK and CK-0 treatments with other fertilization treatments and to examine the alterations in bacterial community structure during the growth period of the summer season cabbage. The PC1 contribution rate more than PC2 (**Figures 4A, B**). The analysis of the **Figure 4A** revealed that the CK, CK-0, A1, B1, and G1 treatments were separated from the C1–F1 treatment on PC1, and the differences were considerable. The C1–F1 treatments altered the bacterial community structure in the soil. The analysis of the **Figure 4B** revealed that the CK and CK-0 treatments were distinctly separated from the A2–G2 treatments on PC1. The observed differences were consistent with the bacterial relative abundance heat map analysis. The A2–G2 treatments altered the bacterial community structure in the soil. In summary, the BC and PGPR application or their combined reduction of nitrogen fertilizers can change the structure of soil bacterial communities.

PCA was conducted to compare CK and CK-0 with other fertilization treatments, examining the alterations in bacterial community structure during the growth period of the winter season cabbage, indicating PC1 contributes more significantly than PC2 (**Figures 4C, D**). The CK, CK-0, E3, F3, and G3 treatments were distinctly separated from the A3–D3 treatments on PC1. The differences were considerable, showing that the A3–D3 treatments altered the bacterial community structure in the soil (**Figure 4C**). The CK and CK-0 treatments were distinctly separated from the A4–G4 treatments on PC1, and the differences were considerable and consistent with the heatmap analysis of bacterial relative abundance. The A4–G4 treatments altered the bacterial community structure in the soil (**Figure 4D**). In summary, the BC and PGPR applications or their combined reduction of nitrogen fertilizers altered the structure of soil bacterial communities, but the enhancement effect of PGPR is significantly strong.

### 3.3 Effect of different fertilization treatments on Chinese cabbage yield

**Figure 5:**
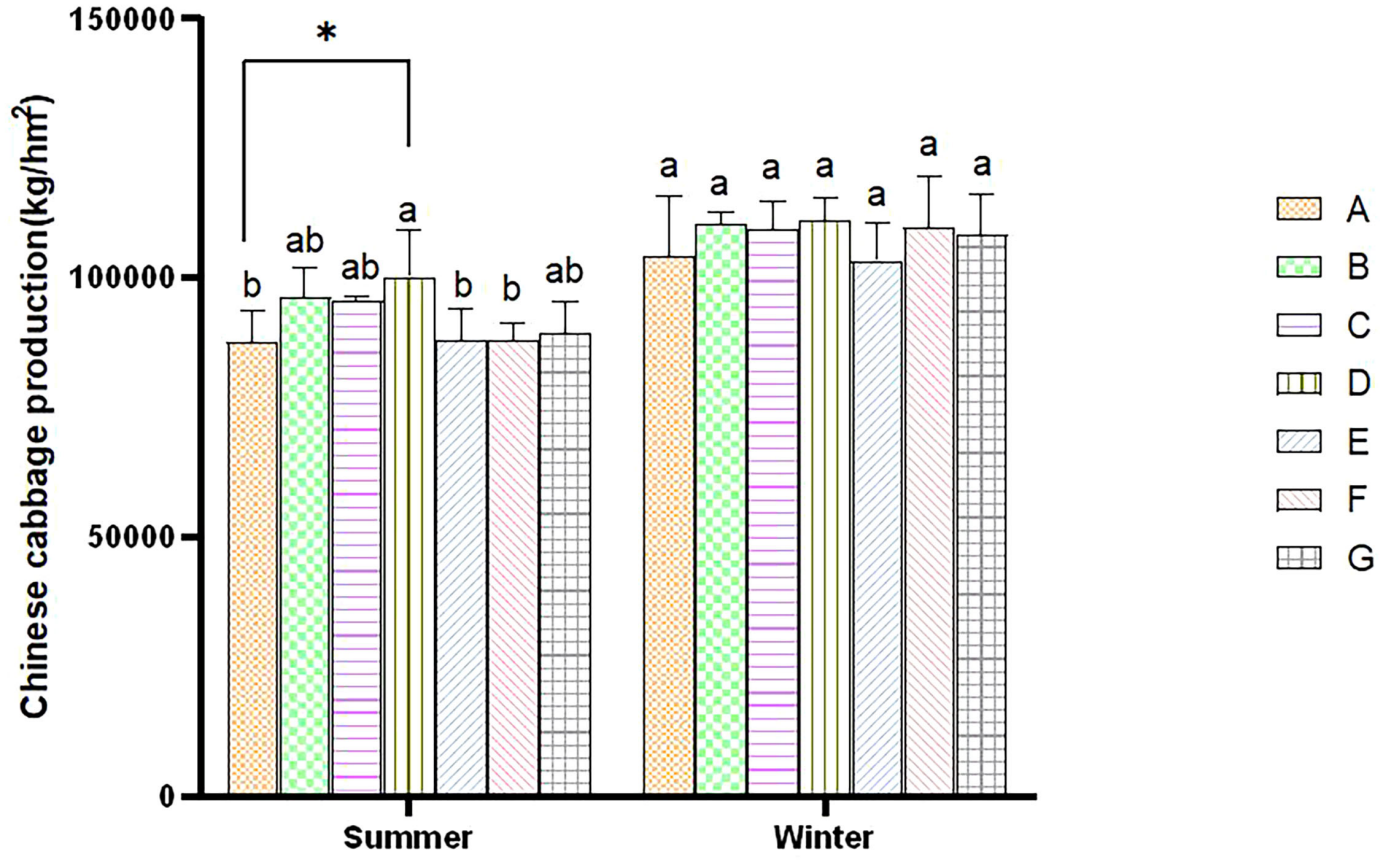
Comparative analysis of cabbage yield within the same season. Error bars refer to the average value ± SD from three biological replicates. Different letters above the columns indicate significant differences (P<0.05). * indicates significance differences at the P<0.05 level.

The yield of cabbage planted in winter was higher than that grown in summer due to climate differences. During the summer, the yield of all treatments increased compared to control A, with D exhibiting the significantly highest yield. The B, C, and G treatments followed in order of yield. Still, there were no significant differences among the three treatments. The yield of the E and F treatments was the lowest, with no significant difference between them. During the winter, the D treatment reached the highest yield compared to control A, and the E treatment reached the lowest yield during winter (**Figure 5**). The combined BC and PGPR applications significantly enhanced the yield of Chinese cabbage. At the same time, such a combined application reduced the application of nitrogen fertilizer without decreasing the yield.

## 4 Discussion

### 4.1 Effect of fertilization level on soil physicochemical properties

The physicochemical properties are the evaluation index of soil quality. Wei et al. (2023) found that the soil pH of the BC application treatment was 7.0% higher than that of the control. This result was also observed here (**Table 2**). The BC application and the reduction of nitrogen fertilizer with PGPR in this study significantly increased soil pH. The mechanism of BC induce soil pH change depends on the alkaline ions and surface functional groups of it. Additionally, the increase in pH can constrain the mobility of heavy metals, thereby ensuring soil health (Pan et al., 2021). The BC application with reduced nitrogen fertilizer, the sole PGPR application, and the combined BC and PGPR application significantly increased the soil OM content (**Table 2**). This result is consistent with the research results found by Urooj et al. (2022). Such an increase in OM content occurs because BC is rich in organic carbon. Biochar application adds exogenous organic carbon to the soil, increasing the carbon pool and OM content and affecting soil fertility.

Alkaline hydrolyzable nitrogen can reflect the nitrogen supply capacity of the soil for the current or recent season. Alkaline hydrolyzable nitrogen is a crucial indicator of soil fertility and is critical for balancing fertilization and effectively controlling agricultural non-point source pollution. Both BC and PGPR enhanced the AH-N content in the soil (**Table 2**). This result is consistent with the research results of Lalay et al. (2022). This result may be attributed to BC enhancing the biological nitrogen fixation capacity and reducing soil N_2_O emissions (Harter et al., 2014). Moreover, PGPR can achieve nitrogen fixation, phosphorus solubilization, potassium release, and other functions by producing nitrogenase, phosphatase, nuclease, or organic acids through metabolism, thereby improving the AH-N, AP, and AK contents and soil fertility. The combination of nitrogen fertilizer reduction and PGPR and their combined use with BC significantly increased the AK content in the soil (**Table 2**). This result may be because the PGPR used in this experiment is a growth-promoting strain with the ability to dissolve phosphorus and potassium, which is conducive to activating potassium in the soil, promoting plant absorption, and thus increasing the content of AK in the soil (Olaniyan et al., 2022). Sui et al. (2022) have noted that adding BC and reducing nitrogen fertilizer application with BC can enhance the AP content in the soil. This result is consistent with this study (**Table 2**), possibly because BC can directly interact with the applied chemical fertilizers, adsorbing phosphate within its porous structure, resulting in a slow loss of effective phosphorus from the soil (Yang et al., 2021).

### 4.2 Effect of fertilization level on soil microbial community diversity

The changes of microorganisms in soil can impact plant growth, and the development of plants may also influence the variations in soil microorganisms (Singh et al., 2022). This study utilized Illumina sequencing technology to demonstrate that different fertilizer treatments significantly impacted the structure of rhizosphere microbial communities in cabbage. The distribution characteristics of the dominant bacteria at the phyla and genera levels in the soil samples in this experiment were analyzed. *Proteobacteria*, *Chloroflexi*, *Firmicutes*, *Bacteroidetes*, and *Actinobacteria* were the dominant abundant bacteria in each treatment (**Figure 1**). These microflora organisms have also been identified as dominant populations in several other studies (Jin et al., 2022; Yin et al., 2022). This result indicated that the soil rhizosphere of the different treatments in this experiment had a common composition of dominant soil microbial communities, but the proportions were different.

Research has shown that *Proteobacteria* are primarily facultative or obligate anaerobes and heterotrophic microorganisms, and that *Sphingomonas* can remove refractory pollutants (Wang et al., 2024), *Bradyrhizobium* has a bioremediation function on soil (Harter et al., 2017). The relative abundance of *Proteobacteria* in the BC and PGPR combined applications was the highest in this study (**Figures 2**, **3**). This result indicated that beneficial microbial communities in the rhizosphere soil were greater than in the other treatments. The ability to remove soil pollutants and repair soil was also the strongest, and environmental friendliness was also the highest (Saeed et al., 2022). *Actinomycetes* can decompose cellulose and lignin, and abundant *Actinomycetes* are beneficial to decomposing plant organic residues in soil (Zhang et al., 2021). In this study, the relative abundance of *Actinomycetes* in the combined BC and PGPR application was the highest (**Figures 2**, **3**), indicating that the OM content in the rhizosphere soil was high, and the soil fertility was the strongest under this treatment condition. This confirmed that the addition of BC and PGPR can indeed improve soil fertility. In addition, some *Firmicutes* can repair their environments, degrade or transform pollutants, and reduce the impact of soil pollution on the ecological environment. *Bacillus* in *Firmicutes* are a widely used biocontrol bacteria with long survival periods and strong resistance. Many strains of these genera have attracted widespread attention from researchers due to their nitrogen-fixing ability, resistance to pathogenic bacteria, and growth-promoting effects on plants (Li et al., 2020; Sun et al., 2022). The relative abundance of *Firmicutes* in the single PGPR application in this study was the highest, followed by the combined BC and PGPR application with reduced nitrogen fertilizer application (**Figures 2**, **3**). This result indicated that both treatments have high environmental friendliness, strong resistance to pathogens, and nitrogen-fixing capability. *Bacteroidetes* participate in the nitrogen cycling process in soil, influencing the content of mineralized nitrogen through its activities. The abundance of *Bacteroidetes* is also high in environments with a high OM content in the soil. In addition, the microbial *Bacteroidetes* community may help enhance plant defense capabilities and reduce disease occurrence (Pan et al., 2023). The relative abundance of *Bacteroidetes* in this study was highest in the single BC or PGPR application (**Figures 2**, **3**), indicating that the two treatments had strong nitrogen-fixing ability and increased soil OM content and disease resistance. In summary, the application of BC and PGPR can effectively increase the relative abundance of beneficial microbial communities in soil, especially biocontrol bacteria. They are direct indicators of root diseases in Chinese cabbage. For example, cabbage clubroot disease caused by *Plasmodiophora brassicae* (Javed et al., 2023). It implied that the application of BC and PGPR could reduce the occurrence of root diseases in Chinese cabbage, but more evidence is needed.

The alpha and beta diversity analyses showed differences in the richness and diversity of rhizosphere soil bacterial communities under different treatments (**Table 3**; **Supplementary Table 8**; **Figure 4**). Compared to conventional fertilization, the application of single BC, single PGPR, and a combination of BC and PGPR significantly increased both the Shannon and Simpson indices of bacteria in the soil (**Table 3**), indicating that the microbial diversity and species dominance of these three treatments were significantly increased, which was consistent with the results of previous studies (Gulnaz et al., 2017; Di Salvo et al., 2018). The differences in the microbial community structure may be attributed to significant differences in environmental factors, as soil physicochemical properties have significant impacts on the microbial community structure. For example, Wang et al. (2021) found that soil physicochemical properties were determinant factors driving changes in the number of soil microorganisms of Gastrodia elata. Another reason may be that different fertilizations lead to different degrees of enrichment of microbial communities. The above showed that soil physicochemical properties and fertilizers were the key factors that produce differences in the microbial community structures.

### 4.3 Effect of fertilization level on Chinese cabbage yield

Dry matter is the final form of crop photosynthetic products, and it guarantees an increased crop yield. Dry matter accumulation is closely related to nitrogen supply at different growth stages. The application of nitrogen fertilizer is irreplaceable in crop production and yield enhancement. The cabbage yield in the three treatments with a 50% nitrogen reduction did not significantly decrease compared to the control (**Figure 5**). This result is consistent with previous research results. Fan et al. (2024) showed that reducing nitrogen between 54% and 80% will not significantly impact the yield of vegetables grown in Southwest China. Therefore, optimizing nitrogen fertilizer application strategies and technologies can maintain or increase vegetable yield, significantly improve nitrogen use efficiency (NUE), and reduce crop nitrogen loss. Our research also showed that BC addition could increase the yield of Chinese cabbage (**Figure 5**), consistent with the results of Tang et al. (2022). At the same time, PGPR has been proven to promote plant growth (Vejan et al., 2016). In this experiment, the application of PGPR also promoted increased cabbage yield, consistent with previous research findings (Gul et al., 2023).

Our study confirmed that a co-application of BC and PGPR can positively affect the Chinese cabbage yield, and even a 50% nitrogen reduction did not lower the Chinese cabbage output. Therefore, more extensive exploration of the optimal application scheme of BC and PGPR at different scenarios and crops needs to be further studied.

## 5 Conclusion

The BC application combined with PGPR significantly increased the yield of cabbage, enhanced soil fertility, optimized the microbial community structure, and increased the bacterial community diversity and relative abundance of potential growth-promoting bacteria (e.g., *Sphingomonas*, *Bradyrhizobium*, *Bacillus*, and *Pseudomonas*). A 50% nitrogen reduction did not significantly impact the above indicators.

In summary, BC can improve the colonization ability and effectiveness of PGPR in plant growth and development and may be an appropriate carrier for PGPR. Combining BC and PGPR could synergistically affect crop yield, soil fertility amelioration, and microbial community optimization. Our research provides a practical case for efficient fertilizer application and chemical fertilizer pollution reduction in crop production.

## Data Availability

The data presented in the study are deposited in the Figshare repository, accession number 10.6084/m9.figshare.27932247.
