# Supplementary material for: The synergistic interaction effect between biochar and plant growth-promoting rhizobacteria on beneficial microbial communities in soil
Source: Front Plant Sci. 2024 Dec 19;15:1501400. doi: 10.3389/fpls.2024.1501400 (PMC11693716; doi:10.3389/fpls.2024.1501400)
Supplement: Supplementary file 1 [file DataSheet1.docx]

Supplementary Material

# Supplementary Figures and Tables

## Supplementary Figures


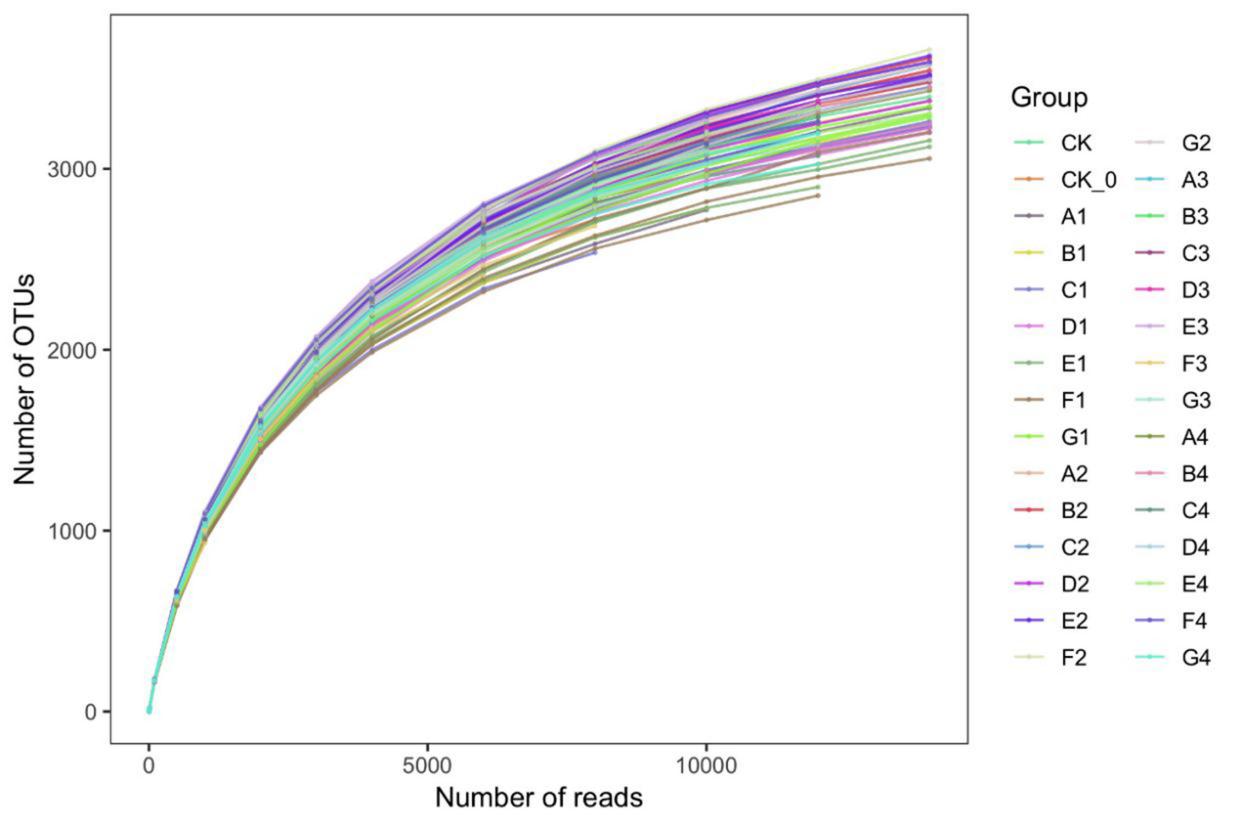


**Supplementary Figure 1.** OTUs curve derived from individual treatment bacterial populations.

Note: CK: sample taken before cabbage planting, blank control; CK–0: microbial agents were added to cabbage before planting, blank control; A1–G1: mid-growth sampling of summer season Chinese cabbage; A2–G2: sampling at the later growth stage of the summer season Chinese cabbage; A3–G3: mid-growth sampling of winter season Chinese cabbage; A4–G4: sampling at the later growth stage of the winter season Chinese cabbage.

## Supplementary Tables

**Supplementary Table 1.** Fertilizers used in the field experiment.

| Fertilizer | Nutrient content (%) | Sources |
| --- | --- | --- |
| Urea | N 46 | Yunnan Yuntianhua Co., Ltd. |
| Compound fertilizer | N∶P_2_O_5_∶K_2_O=18-5-25 | Xiangyun County Hengfeng Fertilizer Co., Ltd. |
| Water-soluble compound fertilizer | N∶P_2_O_5_∶K_2_O=20-20-20 |  |
| Mr Faster 1 | N∶P_2_O_5_∶K_2_O=12-16-10 | Yunnan Weixin Agricultural Science and Technology Co., Ltd. |
| Mr Faster 2 | N∶P_2_O_5_∶K_2_O=19-5-14 |  |
| Mr Faster 3 | N∶P_2_O_5_∶K_2_O=8-4-27 |  |

**Supplementary Table 2.** PGPR agent information.

| Biological agents | Functional strains | Effective bacterial number  (one hundred million/g) | Sources |
| --- | --- | --- | --- |
| PGPR | *Paenibacillus polymyxa* | 2.00 | Dezhou Chuangdi Microbial Resources Co., Ltd. |
|  | *Streptomyces albogriseolus* | 0.01 |  |
|  | *Streptomyces tendae* |  |  |
|  | *Bacillus amyloliquefaciens* | 0.50 |  |
|  | *Bacillus subtilis* |  |  |
|  | *Bacillus pumilus* |  |  |

**Supplementary Table 3.** Fertilization scheme and amount for treatments.

| Treatment | Fertilization time | Type | Application rate (kg/hm^2^) | Method |
| --- | --- | --- | --- | --- |
| A, B, C, D  (The summer season) | After planting cabbage for 7 days | urea | 300 | Hole application |
|  | After planting cabbage for 14 days | compound fertilizer | 300 |  |
|  | After planting cabbage for 20 days | compound fertilizer | 525 |  |
|  | After planting cabbage for 27 days | Water-soluble compound fertilizer | 300 | Irrigation application  fertilizer:water=1:200 |
| A, B, C, D  (The winter season) | After sowing cabbage for 30 days | urea | 300 | Same as the summer season |
|  | After sowing cabbage for 40 days | compound fertilizer | 300 |  |
|  | After sowing cabbage for 50 days | compound fertilizer | 525 |  |
|  | After sowing cabbage for 60 days | Water-soluble compound fertilizer | 300 |  |
| E, F, G  (The summer season) | After planting cabbage for 7 days | Mr Faster 1 | 120 | Mix the fertilizer in proportion (1:200), and then supply water and fertilizer simultaneously through a dropper pipeline |
|  | After planting cabbage for 14 days | Mr Faster 1 & 2 | 90 & 150 |  |
|  | After planting cabbage for 20 days | Mr Faster 2 | 240 |  |
|  | After planting cabbage for 27 days | Mr Faster 2 & 3 | 300 & 210 |  |
| E, F, G  (The winter season) | After sowing cabbage for 30 days | Mr Faster 1 | 120 |  |
|  | After sowing cabbage for 40 days | Mr Faster 1& 2 | 90 & 150 |  |
|  | After sowing cabbage for 50 days | Mr Faster 2 | 240 |  |
|  | After sowing cabbage for 60 days | Mr Faster 2& 3 | 300 & 210 |  |

Note: When E, F, and G were fertilized, A, B, C, and D were sprayed with the same amount of water.

**Supplementary Table 4.** The classification of bacteria in the mid-growth of summer season Chinese cabbage.

| Phylum | Genus | A | B | C | D | E | F | G |
| --- | --- | --- | --- | --- | --- | --- | --- | --- |
| Proteobacteria | *SpHingomonas* | 0.084818a | 0.073149ab | 0.071245ab | 0.069639ab | 0.077443ab | 0.074082ab | 0.063571b |
|  | *Lysobacter* | 0.037564ab | 0.043557a | 0.035790ab | 0.034931ab | 0.032504ab | 0.031963b | 0.033120ab |
|  | *Thermomonas* | 0.014264b | 0.018334ab | 0.020649a | 0.016840ab | 0.016766ab | 0.015627ab | 0.016262ab |
|  | *Erythrobacter* | 0.013536a | 0.013032a | 0.013200a | 0.013349a | 0.013088a | 0.012490a | 0.011762a |
|  | *Ellin6067* | 0.006908b | 0.008775ab | 0.009223ab | 0.009503ab | 0.009671ab | 0.008850ab | 0.010922a |
|  | *Luteimonas* | 0.006142b | 0.007449b | 0.016934a | 0.009204b | 0.009223b | 0.008682b | 0.006852b |
|  | *Pseudomonas* | 0.005545a | 0.008756a | 0.006404a | 0.009242a | 0.006739a | 0.005060a | 0.009111a |
|  | *MND1* | 0.006385b | 0.006777ab | 0.006460b | 0.006889ab | 0.006236b | 0.005470b | 0.009111a |
|  | *Arenimonas* | 0.005582a | 0.007039a | 0.006572a | 0.005844a | 0.007505a | 0.005508a | 0.007860a |
|  | *Nitrosospira* | 0.005844bc | 0.010380a | 0.009391ab | 0.008682ab | 0.002278cd | 0.001755d | 0.003081cd |
|  | *Massilia* | 0.005004a | 0.005059a | 0.006068a | 0.008700a | 0.004716a | 0.004892a | 0.003715a |
|  | *Ellin6055* | 0.005246a | 0.005116a | 0.006105a | 0.005358a | 0.006497a | 0.004443a | 0.004854a |
|  | *Actinobacillus* | 0.004723a | 0.006049a | 0.004499a | 0.004425a | 0.005004a | 0.005769a | 0.005526a |
|  | *Escherichia-Shigella* | 0.004854a | 0.004854a | 0.004406a | 0.004387a | 0.005302a | 0.004836a | 0.004723a |
|  | *Altererythrobacter* | 0.004238a | 0.004462a | 0.004369a | 0.004145a | 0.005899a | 0.005172a | 0.003958a |
|  | *Pseudoxanthomonas* | 0.003323a | 0.004182a | 0.004070a | 0.003734a | 0.003155a | 0.003361a | 0.004499a |
|  | *Reyranella* | 0.004667a | 0.002539b | 0.001587b | 0.002352b | 0.002632b | 0.002016b | 0.002352b |
|  | *Mesorhizobium* | 0.002147a | 0.002782a | 0.002520a | 0.002352a | 0.002987a | 0.001811a | 0.002352a |
|  | *Nitrobacter* | 0.002949abc | 0.003622a | 0.003062ab | 0.002240bcd | 0.001792cd | 0.001270d | 0.001382d |
|  | *Aquicella* | 0.001886a | 0.001568a | 0.001811a | 0.001550a | 0.001792a | 0.002763a | 0.002726a |
|  | *Klebsiella* | 0.001755a | 0.001923a | 0.002539a | 0.001979a | 0.002072a | 0.001624a | 0.001774a |
|  | *Rhizobacter* | 0.001755ab | 0.001774ab | 0.001755ab | 0.002334a | 0.001829ab | 0.001774ab | 0.001494b |
|  | *Allorhizobium-Neorhizobium-Pararhizobium-Rhizobium* | 0.001587a | 0.001979a | 0.002315a | 0.002072a | 0.002091a | 0.001494a | 0.001587a |
| Firmicutes | *Clostridium*  *sensu stricto 1* | 0.023132a | 0.024271a | 0.022609a | 0.024346a | 0.023673a | 0.026082a | 0.024234a |
|  | *Lactobacillus* | 0.012901a | 0.012602a | 0.015235a | 0.012826a | 0.011837a | 0.014824a | 0.014488a |
|  | *Lachnospiraceae NK4A136 group* | 0.007580b | 0.007823b | 0.007169b | 0.006628b | 0.008420ab | 0.010959a | 0.008495ab |
|  | *Ruminiclostridium 9* | 0.004705a | 0.004742a | 0.004163a | 0.004014a | 0.004630a | 0.005209a | 0.004014a |
|  | *Ruminococcaceae UCG-002* | 0.002558a | 0.002632a | 0.003417a | 0.002950a | 0.002782a | 0.003118a | 0.003249a |
|  | *Bacillus* | 0.002390a | 0.002296a | 0.002502a | 0.003043a | 0.002632a | 0.002913a | 0.002576a |
|  | *Ruminococcus 1* | 0.002390a | 0.002128a | 0.001848a | 0.001829a | 0.002147a | 0.002744a | 0.002091a |
|  | *Intestinibacter* | 0.001815a | 0.001729a | 0.001791a | 0.001791a | 0.001889a | 0.002038a | 0.001717a |
| Acidobacteria | *Subgroup 10* | 0.008364a | 0.008401a | 0.007748a | 0.008513a | 0.008196a | 0.008719a | 0.008476a |
|  | *RB41* | 0.006441a | 0.005452a | 0.005041a | 0.005974a | 0.005806a | 0.005844a | 0.006422a |
|  | *Candidatus Solibacter* | 0.002296a | 0.001550ab | 0.002371a | 0.002147ab | 0.001867ab | 0.001718ab | 0.001400b |
|  | *JGI 0001001-H03* | 0.002016a | 0.001494a | 0.001400a | 0.001755a | 0.001979a | 0.001923a | 0.001904a |
|  | *Renibacterium* | 0.004257c | 0.004630c | 0.006534bc | 0.004798c | 0.006273bc | 0.012322ab | 0.014917a |
|  | *Rhodococcus* | 0.005601a | 0.005508a | 0.005302a | 0.005918a | 0.006759a | 0.006534a | 0.006703a |
|  | *Streptomyces* | 0.003678a | 0.003043a | 0.003977a | 0.003883a | 0.004294a | 0.003697a | 0.003883a |
|  | *Nocardioides* | 0.002987a | 0.002576a | 0.004742a | 0.003193a | 0.003827a | 0.002763a | 0.003865a |
|  | *Aeromicrobium* | 0.001419a | 0.001195a | 0.002184a | 0.001624a | 0.002539a | 0.001624a | 0.002166a |
| Bacteroidetes | *Flavisolibacter* | 0.006179a | 0.006703a | 0.007225a | 0.007972a | 0.006572a | 0.005844a | 0.006460a |
|  | *Bacteroides* | 0.001568b | 0.001550b | 0.001979ab | 0.002240ab | 0.002147ab | 0.002334a | 0.001811ab |
|  | *Nitrospira* | 0.006217a | 0.005078a | 0.005078a | 0.004518a | 0.004331a | 0.004033a | 0.003622a |
|  | *Helicobacter* | 0.004070a | 0.004070a | 0.003958a | 0.004854a | 0.004387a | 0.005172a | 0.005265a |
| Verrucomicrobia | *Luteolibacter* | 0.003137a | 0.002558a | 0.003118a | 0.002464a | 0.002614a | 0.001606a | 0.003846a |
|  | *LacunispHaera* | 0.001960a | 0.001867a | 0.002688a | 0.002408a | 0.002184a | 0.001886a | 0.003081a |
|  | *Pirellula* | 0.004630a | 0.003305ab | 0.002464b | 0.002763b | 0.002446b | 0.002595b | 0.002819b |
|  | *OLB13* | 0.003062a | 0.002651a | 0.002296a | 0.002614a | 0.002315a | 0.001774a | 0.002614a |
|  | *Candidatus Nitrocosmicus* | 0.002744a | 0.002744a | 0.002016a | 0.003566a | 0.001755a | 0.002166a | 0.001904a |
|  | *Mycoplasma* | 0.001214a | 0.001606a | 0.001624a | 0.001531a | 0.001438a | 0.004051a | 0.001400a |

**Supplementary Table 5.** The classification of bacteria in the late growth of summer season Chinese cabbage.

| Phylum | Genus | A | B | C | D | E | F | G |
| --- | --- | --- | --- | --- | --- | --- | --- | --- |
| Proteobacteria | *SpHingomonas* | 0.062636a | 0.062611a | 0.063031a | 0.055671a | 0.056004a | 0.064031a | 0.062228a |
|  | *Lysobacter* | 0.033281ab | 0.026353b | 0.037690a | 0.030552ab | 0.026749b | 0.027304ab | 0.031392ab |
|  | *Thermomonas* | 0.016820a | 0.013078ab | 0.016968a | 0.013473ab | 0.010942b | 0.011831ab | 0.011954ab |
|  | *Pseudomonas* | 0.012683a | 0.010497a | 0.012386a | 0.009744a | 0.009793a | 0.007373a | 0.010497a |
|  | *Erythrobacter* | 0.011275a | 0.009892a | 0.010373a | 0.010065a | 0.009250a | 0.011250a | 0.010077a |
|  | *Luteimonas* | 0.012362a | 0.011880a | 0.008768ab | 0.010114ab | 0.007348b | 0.008595ab | 0.007620b |
|  | *Ellin6067* | 0.007731a | 0.009287a | 0.008595a | 0.010040a | 0.009188a | 0.008076a | 0.009472a |
|  | *MND1* | 0.008472a | 0.008719a | 0.008126a | 0.009139a | 0.008904a | 0.008805a | 0.009003a |
|  | *Arenimonas* | 0.009114a | 0.008496a | 0.006681a | 0.009682a | 0.007669a | 0.007002a | 0.008521a |
|  | *Ramlibacter* | 0.005520a | 0.005545a | 0.005804a | 0.006064a | 0.005458a | 0.005174a | 0.005273a |
|  | *Actinobacillus* | 0.005125a | 0.005681a | 0.004754a | 0.004940a | 0.005384a | 0.004594a | 0.004384a |
|  | *Ellin6055* | 0.004248a | 0.004001a | 0.003853a | 0.004964a | 0.004347a | 0.004545a | 0.004927a |
|  | *Escherichia-Shigella* | 0.004100a | 0.004421a | 0.004347a | 0.004409a | 0.004520a | 0.004754a | 0.003952a |
|  | *Altererythrobacter* | 0.004174a | 0.004421a | 0.004125a | 0.004396a | 0.004125a | 0.004804a | 0.003989a |
|  | *Nitrosospira* | 0.006372a | 0.005841a | 0.005310a | 0.004273a | 0.002136a | 0.002420a | 0.001680a |
|  | *Pseudoxanthomonas* | 0.004619a | 0.003273a | 0.003137a | 0.003631a | 0.003100a | 0.003445a | 0.004038a |
|  | *Bradyrhizobium* | 0.003161ab | 0.003804ab | 0.004446a | 0.003507ab | 0.002902b | 0.002853b | 0.003297ab |
|  | *Methylibium* | 0.003026ab | 0.003643ab | 0.004075a | 0.003371ab | 0.003013ab | 0.002828b | 0.002507b |
|  | *Reyranella* | 0.002556a | 0.003087a | 0.003297a | 0.002964a | 0.003384a | 0.002680a | 0.003964a |
|  | *Aquicella* | 0.002692a | 0.003310a | 0.002667a | 0.003557a | 0.003075a | 0.003112a | 0.002779a |
|  | *Massilia* | 0.003692a | 0.002779a | 0.003087a | 0.003063a | 0.002742a | 0.003013a | 0.002359a |
|  | *SWB02* | 0.002359a | 0.002433a | 0.001729a | 0.002334a | 0.002359a | 0.002717a | 0.002371a |
|  | *Jahnella* | 0.002371a | 0.001865a | 0.002322a | 0.002359a | 0.002334a | 0.001840a | 0.002124a |
|  | *Dokdonella* | 0.002396a | 0.002248a | 0.001420a | 0.002507a | 0.001840a | 0.001778a | 0.001729a |
| Firmicutes | *Clostridium sensu stricto 1* | 0.024032a | 0.025526a | 0.023266a | 0.022908a | 0.025217a | 0.023772a | 0.024427a |
|  | *Lactobacillus* | 0.012954a | 0.013041a | 0.011559a | 0.013757a | 0.013226a | 0.012559a | 0.012275a |
|  | *Lachnospiraceae NK4A136 group* | 0.007694a | 0.007397a | 0.007360a | 0.007545a | 0.007792a | 0.007731a | 0.007113a |
|  | *Ruminiclostridium 9* | 0.004038a | 0.004285a | 0.003902a | 0.004792a | 0.004841a | 0.004829a | 0.004014a |
|  | *Bacillus* | 0.002989ab | 0.003322a | 0.002408ab | 0.002309b | 0.003075ab | 0.002914ab | 0.002507ab |
|  | *Ruminococcaceae UCG-002* | 0.002865ab | 0.003026a | 0.002791ab | 0.002593ab | 0.002643ab | 0.002840ab | 0.002532b |
|  | *Ruminococcus 1* | 0.002173a | 0.002766a | 0.002322a | 0.002198a | 0.002667a | 0.002420a | 0.002433a |
|  | *Intestinibacter* | 0.001815a | 0.001729a | 0.001791a | 0.001791a | 0.001889a | 0.002038a | 0.001717a |
| Actinobacteria | *Renibacterium* | 0.013621a | 0.012782a | 0.017956a | 0.006545a | 0.007274a | 0.007410a | 0.024390a |
|  | *Rhodococcus* | 0.005829a | 0.006335a | 0.005656a | 0.006212a | 0.006335a | 0.006101a | 0.006842a |
|  | *Streptomyces* | 0.004409a | 0.003631a | 0.003680a | 0.004989a | 0.003347a | 0.003408a | 0.004038a |
|  | *Nocardioides* | 0.004508a | 0.003470a | 0.004051a | 0.003322a | 0.003347a | 0.003668a | 0.002927a |
|  | *Aeromicrobium* | 0.002235a | 0.002038a | 0.002087a | 0.002334a | 0.001964a | 0.001889a | 0.001840a |
|  | *Subgroup 10* | 0.008484a | 0.007607a | 0.007928a | 0.009460a | 0.009435a | 0.010546a | 0.009830a |
|  | *RB41* | 0.005347a | 0.005323a | 0.005483a | 0.004829a | 0.005384a | 0.005915a | 0.005199a |
|  | *Candidatus Solibacter* | 0.002235bc | 0.001791c | 0.002470abc | 0.002655abc | 0.002211bc | 0.003087a | 0.002507abc |
| Nitrospirae | *Nitrospira* | 0.005804a | 0.005952a | 0.007681a | 0.007261a | 0.006273a | 0.006743a | 0.005570a |
| Bacteroidetes | *Flavisolibacter* | 0.005471a | 0.004532a | 0.005928a | 0.004446a | 0.004310a | 0.005088a | 0.004322a |
|  | *Bacteroides* | 0.002186ab | 0.001482b | 0.001852ab | 0.001852ab | 0.001815ab | 0.002408a | 0.001680ab |
| Planctomycetes | *Pirellula* | 0.003520a | 0.004310a | 0.004026a | 0.004063a | 0.004656a | 0.004693a | 0.005014a |
| Epsilonbacteraeota | *Helicobacter* | 0.004273a | 0.004717a | 0.003705a | 0.004186a | 0.004112a | 0.003964a | 0.004075a |
| Verrucomicrobia | *Luteolibacter* | 0.003964a | 0.003507a | 0.003643a | 0.003618a | 0.003198a | 0.002890a | 0.005187a |
|  | *LacunispHaera* | 0.003075a | 0.003260a | 0.003458a | 0.002964a | 0.002371a | 0.002507a | 0.002840a |
|  | *Chthoniobacter* | 0.001655a | 0.001568a | 0.001828a | 0.001433a | 0.001803a | 0.002013a | 0.001877a |
| Thaumarchaeota | *Candidatus Nitrocosmicus* | 0.002161b | 0.002692ab | 0.003495ab | 0.002729ab | 0.002680ab | 0.003939a | 0.002495ab |
| Chloroflexi | *OLB13* | 0.002458ab | 0.002173b | 0.002840ab | 0.001988b | 0.002025b | 0.003223a | 0.001926b |

**Supplementary Table 6.** The classification of bacteria in the mid-growth of winter season Chinese cabbage.

| Phylum | Genus | A | B | C | D | E | F | G |
| --- | --- | --- | --- | --- | --- | --- | --- | --- |
| Proteobacteria | *SpHingomonas* | 0.049952ab | 0.042267cd | 0.04707bc | 0.052257ab | 0.047038bc | 0.054339a | 0.040506d |
|  | *Lysobacter* | 0.0255522a | 0.018444d | 0.023215bc | 0.021742c | 0.017259d | 0.024944ab | 0.02251c |
|  | *Thermomonas* | 0.010695a | 0.006308c | 0.008453b | 0.00999ab | 0.009094b | 0.008902b | 0.00554c |
|  | *Altererythrobacter* | 0.006340bcd | 0.004835d | 0.006724bcd | 0.005251cd | 0.009062a | 0.007269b | 0.006884bc |
|  | *Luteimonas* | 0.006180b | 0.005443b | 0.01172a | 0.006692b | 0.006820b | 0.005091bc | 0.003650c |
|  | *Pseudomonas* | 0.005828bc | 0.004195c | 0.006948ab | 0.005604bc | 0.005828bc | 0.005219bc | 0.007749a |
|  | *MND1* | 0.004227d | 0.008229a | 0.005700bcd | 0.006212bc | 0.004579d | 0.004803cd | 0.006980ab |
|  | *Ellin6067* | 0.004611a | 0.006372a | 0.005187a | 0.004611a | 0.004899a | 0.004867a | 0.006308a |
|  | *Psychrobacter* | 0.003842abc | 0.004419abc | 0.005187ab | 0.005540a | 0.004515abc | 0.003170c | 0.003490bc |
|  | *Arenimonas* | 0.004387bc | 0.004259bc | 0.003490c | 0.004515bc | 0.005732ab | 0.004675bc | 0.007077a |
|  | *Ectothiorhodospira* | 0.005123a | 0.003362b | 0.004035ab | 0.004611ab | 0.003394b | 0.004259ab | 0.003490ab |
| Proteobacteria | *Ramlibacter* | 0.003458bc | 0.005027a | 0.002498c | 0.003714b | 0.003714b | 0.003394bc | 0.003971b |
|  | *Massilia* | 0.005059a | 0.003202bc | 0.003234bc | 0.003522b | 0.002498c | 0.003266bc | 0.002914bc |
|  | *Escherichia-Shigella* | 0.003202a | 0.00349a | 0.002594a | 0.003362a | 0.003426a | 0.003362a | 0.003554a |
|  | *Nitrosospira* | 0.006852a | 0.001921c | 0.005027b | 0.003971b | 0.001633c | 0.002081c | 0.001153c |
|  | *Reyranella* | 0.002370b | 0.003586a | 0.002305b | 0.001857b | 0.001889b | 0.002209b | 0.002722b |
|  | *Pseudoxanthomonas* | 0.001953ab | 0.002658a | 0.001729b | 0.002434ab | 0.002370ab | 0.001921ab | 0.002081ab |
|  | *Desulfovibrio* | 0.002241a | 0.001921ab | 0.001185b | 0.002434a | 0.001921ab | 0.001921ab | 0.001633ab |
|  | *Actinobacillus* | 0.003074a | 0.002434abc | 0.001569c | 0.001793bc | 0.002434abc | 0.001889bc | 0.002786ab |
|  | *Aquicella* | 0.001633bc | 0.002914a | 0.001697bc | 0.001953bc | 0.001025c | 0.001761bc | 0.002370ab |
|  | *Devosia* | 0.001793a | 0.001633a | 0.002177a | 0.002049a | 0.002209a | 0.002049a | 0.002081a |
| Firmicutes | *Lachnospiraceae NK4A136 group* | 0.011175ab | 0.009318b | 0.012808a | 0.013000a | 0.010631ab | 0.012008a | 0.010727ab |
|  | *Lactobacillus* | 0.010054b | 0.011015b | 0.014249a | 0.009734b | 0.009062b | 0.011111b | 0.009382b |
|  | *Clostridium sensu stricto 1* | 0.014025a | 0.011655ab | 0.010375b | 0.009254b | 0.012040ab | 0.008646b | 0.010407ab |
|  | *Ruminiclostridium 9* | 0.003971bc | 0.003746bcd | 0.003490cd | 0.004707ab | 0.005572a | 0.005443a | 0.002754d |
|  | *Bacillus* | 0.002177bc | 0.002337abc | 0.002850a | 0.002466ab | 0.002882a | 0.002882a | 0.001729c |
|  | *Ruminococcus 1* | 0.002081b | 0.002209ab | 0.002882a | 0.001281c | 0.001761bc | 0.001953bc | 0.002370ab |
|  | *Ruminococcaceae UCG-002* | 0.001953bc | 0.00237ab | 0.002690a | 0.001857c | 0.001761c | 0.001793c | 0.001473c |
| Actinobacteria | *Renibacterium* | 0.006564c | 0.009990c | 0.016362b | 0.001857d | 0.001057d | 0.002434d | 0.024015a |
|  | *Nocardioides* | 0.002498bc | 0.001953c | 0.002273bc | 0.003138b | 0.004675a | 0.003170b | 0.002209bc |
|  | *Rhodococcus* | 0.003298ab | 0.003874a | 0.002466b | 0.002818ab | 0.002818ab | 0.002850ab | 0.003074ab |
|  | *Streptomyces* | 0.002177abc | 0.003074a | 0.002337abc | 0.002722ab | 0.001633c | 0.001761bc | 0.001985bc |
|  | *Subgroup 10* | 0.007365a | 0.005411b | 0.005251b | 0.006756a | 0.00698a | 0.008005a | 0.006948a |
|  | *RB41* | 0.005732a | 0.005219ab | 0.005347a | 0.003362c | 0.003874bc | 0.005091ab | 0.005187ab |
|  | *Candidatus Solibacter* | 0.001409b | 0.001377b | 0.001377b | 0.002049b | 0.001441b | 0.003074a | 0.002113b |
| Bacteroidetes | *Flavisolibacter* | 0.004931a | 0.003298bc | 0.003682bc | 0.003458bc | 0.003106c | 0.003939bc | 0.004259ab |
|  | *Bacteroides* | 0.002882cd | 0.002049e | 0.005668a | 0.002594de | 0.004323b | 0.003554bc | 0.002466de |
|  | *Prevotellaceae UCG-004* | 0.002273bc | 0.002690b | 0.003650a | 0.001665c | 0.002337bc | 0.001569c | 0.001825c |
| Verrucomicrobia | *Luteolibacter* | 0.002273bc | 0.002754b | 0.001953bc | 0.000736c | 0.003202b | 0.002241bc | 0.005219a |
|  | *LacunispHaera* | 0.001313c | 0.001665bc | 0.002273ab | 0.001729abc | 0.002562a | 0.002241ab | 0.002017abc |
|  | *Chthoniobacter* | 0.001985ab | 0.002498a | 0.001857ab | 0.001889ab | 0.001409b | 0.002273ab | 0.001793ab |
| Planctomycetes | *Pirellula* | 0.003266a | 0.004099a | 0.004003a | 0.003362a | 0.004227a | 0.003266a | 0.002882a |
|  | *Pir4 lineage* | 0.001889ab | 0.002594a | 0.001313b | 0.002273ab | 0.002850a | 0.002562a | 0.001857ab |
|  | *Gemmata* | 0.003010a | 0.001729bc | 0.001953bc | 0.001441bc | 0.001057c | 0.002177ab | 0.001313bc |
| Nitrospirae | *Nitrospira* | 0.004963a | 0.004835a | 0.005796a | 0.004643a | 0.002370b | 0.005251a | 0.002434b |
|  | *Leptospirillum* | 0.002145a | 0.001985a | 0.002337a | 0.002209a | 0.002113a | 0.002466a | 0.002049a |
| Thaumarchaeota | *Candidatus Nitrocosmicus* | 0.014281a | 0.010599bc | 0.011880ab | 0.014569a | 0.010759bc | 0.007621cd | 0.006180d |
|  | *Candidatus NitrosospHaera* | 0.002946a | 0.001921a | 0.002370a | 0.002530a | 0.003266a | 0.001825a | 0.003394a |
| Chloroflexi | *OLB13* | 0.007621b | 0.007557b | 0.008197b | 0.008453b | 0.017099a | 0.009286b | 0.007205b |
| Epsilonbacteraeota | *Helicobacter* | 0.009638ab | 0.008069b | 0.010759a | 0.008838ab | 0.009030ab | 0.008421b | 0.009062ab |

**Supplementary Table 7.** The classification of bacteria in the late growth of winter season Chinese cabbage.

| Phylum | Genus | A | B | C | D | E | F | G |
| --- | --- | --- | --- | --- | --- | --- | --- | --- |
| Proteobacteria | *SpHingomonas* | 0.045161c | 0.050791b | 0.055151a | 0.052524ab | 0.055498a | 0.055815a | 0.053072ab |
|  | *Lysobacter* | 0.025930c | 0.030174bc | 0.035516a | 0.027807bc | 0.026623c | 0.032282ab | 0.032311ab |
|  | *Thermomonas* | 0.014928a | 0.015188a | 0.015073a | 0.010568b | 0.010742b | 0.014091a | 0.011348b |
|  | *Altererythrobacter* | 0.005602c | 0.008720b | 0.010193ab | 0.010915ab | 0.011435a | 0.010106ab | 0.011348a |
|  | *Pseudomonas* | 0.00667c | 0.007767c | 0.011117ab | 0.009760b | 0.007017c | 0.006815c | 0.012301a |
|  | *Arenimonas* | 0.005198d | 0.006237d | 0.009327b | 0.011348a | 0.008056c | 0.007825c | 0.012070a |
|  | *MND1* | 0.009153ab | 0.009673a | 0.008085bc | 0.007854bc | 0.007219c | 0.007941bc | 0.007941bc |
|  | *Ellin6067* | 0.008201a | 0.008518a | 0.00641b | 0.007046ab | 0.008201a | 0.007450ab | 0.007074ab |
|  | *Ellin6055* | 0.006179a | 0.005775a | 0.006324a | 0.007392a | 0.007074a | 0.006555a | 0.007132a |
|  | *Ramlibacter* | 0.004418c | 0.004736c | 0.006179a | 0.005515ab | 0.004620c | 0.004591c | 0.005053bc |
|  | *Luteimonas* | 0.005977a | 0.004707b | 0.004938b | 0.003985b | 0.004043b | 0.004158b | 0.002772c |
|  | *Actinobacillus* | 0.004909abc | 0.003840c | 0.005111ab | 0.004736abc | 0.004187bc | 0.004649abc | 0.005688a |
|  | *Massilia* | 0.005544a | 0.004909a | 0.004822ab | 0.004360ab | 0.003119c | 0.003581bc | 0.004447ab |
|  | *Erythrobacter* | 0.002628b | 0.004158a | 0.004476a | 0.003378ab | 0.004389a | 0.004620a | 0.003465ab |
|  | *Escherichia-Shigella* | 0.003927a | 0.003465a | 0.003581a | 0.003840a | 0.003754a | 0.004331a | 0.003956a |
|  | *Reyranella* | 0.003321a | 0.003869a | 0.003350a | 0.003263a | 0.003552a | 0.003407a | 0.003898a |
|  | *Nitrosospira* | 0.006988a | 0.003783b | 0.003898b | 0.001704d | 0.001761d | 0.003263bc | 0.002223cd |
|  | *Pseudoxanthomonas* | 0.002945ab | 0.002108b | 0.003407a | 0.003292a | 0.003176ab | 0.003350a | 0.003696a |
|  | *Aquicella* | 0.00335abc | 0.003812a | 0.003494ab | 0.002079d | 0.002426cd | 0.003234abc | 0.002772bcd |
|  | *Devosia* | 0.002685b | 0.002166b | 0.002916b | 0.002945b | 0.002599b | 0.002714b | 0.003696a |
|  | *Nitrobacter* | 0.002685ab | 0.002685ab | 0.003205a | 0.002426abc | 0.001733c | 0.002108bc | 0.002599ab |
|  | *Mesorhizobium* | 0.001530b | 0.002079ab | 0.002050ab | 0.002974a | 0.002830a | 0.002888a | 0.001646b |
|  | *SWB02* | 0.001733b | 0.001761b | 0.001848ab | 0.003003a | 0.001992a | 0.001473b | 0.002628a |
|  | *Aeromonas* | 0.000520d | 0.001328c | 0.004245a | 0.003003b | 0.000491d | 0.000982cd | 0.003812a |
| Firmicutes | *Clostridium sensu stricto 1* | 0.022147a | 0.022436a | 0.020068b | 0.020588ab | 0.022147a | 0.021137ab | 0.020155b |
|  | *Lactobacillus* | 0.012330a | 0.012532a | 0.010973ab | 0.010424b | 0.011579ab | 0.011579ab | 0.011001ab |
|  | *Lachnospiraceae NK4A136 group* | 0.006959bc | 0.007219bc | 0.008778a | 0.007767abc | 0.008114ab | 0.008229ab | 0.006526c |
|  | *Ruminiclostridium 9* | 0.003754ab | 0.003090b | 0.004274ab | 0.005111a | 0.005111a | 0.003985ab | 0.003090b |
|  | *Bacillus* | 0.002859ab | 0.003090ab | 0.002108c | 0.003609a | 0.002628ab | 0.002252ab | 0.002657ab |
|  | *Ruminococcaceae UCG-002* | 0.003147a | 0.003090a | 0.002830a | 0.002772a | 0.002685a | 0.002685a | 0.002541a |
|  | *Ruminococcus 1* | 0.002108bc | 0.002599ab | 0.002916a | 0.001906c | 0.001761c | 0.002570ab | 0.002223bc |
| Actinobacteria | *Renibacterium* | 0.016632b | 0.011752c | 0.006815d | 0.004678d | 0.003956d | 0.004822d | 0.030636a |
|  | *Rhodococcus* | 0.006381a | 0.006122ab | 0.004505c | 0.004967bc | 0.005429abc | 0.005400abc | 0.005313abc |
|  | *Streptomyces* | 0.004360ab | 0.005053a | 0.004880a | 0.002743c | 0.002657c | 0.003552bc | 0.003638bc |
|  | *Nocardioides* | 0.003003a | 0.003176a | 0.003090a | 0.003436a | 0.002685a | 0.002426a | 0.003234a |
|  | *Aeromicrobium* | 0.001502c | 0.002079abc | 0.002050abc | 0.001646c | 0.002541a | 0.002079abc | 0.002252ab |
|  | *Subgroup 10* | 0.009327a | 0.007074b | 0.007508b | 0.007912b | 0.007132b | 0.007508b | 0.007565b |
|  | *RB41* | 0.006930a | 0.005255b | 0.004967b | 0.004967b | 0.004793b | 0.005053b | 0.004245b |
|  | *Candidatus Solibacter* | 0.001675c | 0.002685b | 0.003581a | 0.002801b | 0.002772b | 0.002888b | 0.002801b |
| Verrucomicrobia | *Luteolibacter* | 0.002483c | 0.003465bc | 0.003898b | 0.004851b | 0.004389b | 0.004764b | 0.008374a |
|  | *LacunispHaera* | 0.002195a | 0.002454a | 0.002685a | 0.002888a | 0.002830a | 0.002483a | 0.002916a |
|  | *Chthoniobacter* | 0.001184b | 0.001559ab | 0.001935ab | 0.001704ab | 0.002195a | 0.002021a | 0.002252a |
| Bacteroidetes | *Flavisolibacter* | 0.003350b | 0.004591a | 0.004562a | 0.002108c | 0.003147b | 0.004274a | 0.003032b |
|  | *Bacteroides* | 0.001819ab | 0.002454a | 0.002021ab | 0.001357b | 0.002310ab | 0.002483a | 0.001877ab |
| Planctomycetes | *Pirellula* | 0.003552a | 0.004129a | 0.004100a | 0.004418a | 0.003494a | 0.004331a | 0.004591a |
|  | *Pir4 lineage* | 0.001213b | 0.001097b | 0.001444ab | 0.002195a | 0.002339a | 0.001617ab | 0.002368a |
| Nitrospirae | *Nitrospira* | 0.006815ab | 0.007421ab | 0.007681a | 0.006179abc | 0.004447c | 0.005891bc | 0.005660bc |
| Chloroflexi | *OLB13* | 0.002454a | 0.002310a | 0.002743a | 0.002628a | 0.001992a | 0.002397a | 0.002166a |
| Epsilonbacteraeota | *Helicobacter* | 0.004447a | 0.003696ab | 0.003840ab | 0.003609ab | 0.003321b | 0.002368c | 0.003812ab |
| Thaumarchaeota | *Candidatus Nitrocosmicus* | 0.003321a | 0.003061a | 0.002223ab | 0.001299b | 0.001328b | 0.002223ab | 0.002743a |

**Supplementary Table 8.** Bacterial diversity index between the same treatment at different times.

| Index | Treatments | 30d | 60d | 115d | 160d |
| --- | --- | --- | --- | --- | --- |
| Observed | A | 943.33c | 1035.00b | 1102.33a | 1070.33ab |
|  | B | 1034.33b | 1029.67b | 1131.33a | 1123.00a |
|  | C | 986.33c | 1034.00b | 1103.00a | 1106.33a |
|  | D | 967.33c | 1020.33b | 1094.00a | 1114.00a |
|  | E | 975.00c | 1066.33b | 1167.00a | 1127.00a |
|  | F | 941.00b | 1018.00b | 946.00b | 1146.67a |
|  | G | 1006.67b | 1014.00b | 1094.33a | 1093.67a |
| Chao1 | A | 1971.46b | 2456.52a | 2612.89a | 2417.82a |
|  | B | 2363.63a | 2370.26a | 2511.65a | 2662.78a |
|  | C | 2162.81b | 2447.25ab | 2730.49a | 2583.75a |
|  | D | 2143.18a | 2155.77a | 2387.58a | 2522.94a |
|  | E | 2021.41b | 2408.25ab | 2745.82a | 2672.02a |
|  | F | 2006.25ab | 2100.30ab | 1731.75b | 2727.46a |
|  | G | 2274.97a | 2278.52a | 2404.12a | 2556.51a |
| Shannon | A | 6.22c | 6.42b | 6.52a | 6.50ab |
|  | B | 6.41b | 6.40b | 6.60a | 6.57a |
|  | C | 6.30c | 6.40b | 6.52a | 6.56a |
|  | D | 6.27c | 6.41b | 6.54a | 6.57a |
|  | E | 6.25c | 6.49b | 6.65a | 6.60a |
|  | F | 6.24c | 6.44b | 6.39b | 6.62a |
|  | G | 6.39b | 6.41b | 6.53a | 6.50a |
| Simpson | A | 0.9954b | 0.9967a | 0.9971a | 0.9970a |
|  | B | 0.9965b | 0.9966b | 0.9975a | 0.9973a |
|  | C | 0.9960c | 0.9966b | 0.9971a | 0.9974a |
|  | D | 0.9958c | 0.9968b | 0.9973a | 0.9975a |
|  | E | 0.9949c | 0.9970b | 0.9976a | 0.9975a |
|  | F | 0.9956c | 0.9969b | 0.9969b | 0.9975a |
|  | G | 0.9966a | 0.9968a | 0.9969a | 0.9967a |
